# Hafnium-Based Ferroelectric Post-Moore Electronics: Device Physics, Integration Architectures, and Neuromorphic System Implementation

**DOI:** 10.1007/s40820-026-02158-z

**Published:** 2026-04-13

**Authors:** Xiangwei Chen, Zheng Wang, Jialin Meng, Tianyu Wang

**Affiliations:** 1 Shandong Key Laboratory of Next-Generation Semiconductor Technology and Systems, School of Integrated Circuits, Shandong University, Jinan, 250100 People’s Republic of China; 2 Shenzhen Research Institute of Shandong University, Shenzhen, 518100 People’s Republic of China; 3 National International Innovation Center, Shanghai, 201203 People’s Republic of China; 4 Key Laboratory of Computational Neuroscience and Brain-Inspired Intelligence (Fudan University), Ministry of Education, Shanghai, 200433 People’s Republic of China; 5 State Key Laboratory of Crystal Materials, Shandong University, Jinan, 250100 People’s Republic of China

**Keywords:** Hafnium oxide, Ferroelectric materials, Neuromorphic computing, Non-volatile memory

## Abstract

Hafnium-based ferroelectric (Hf-FEs) materials overcome the limitations of perovskite ferroelectric materials. Owing to their compatibility with the complementary metal–oxide–semiconductor process and high scalability, Hf-FEs devices have driven the development of non-volatile memory and neuromorphic computing, demonstrating significant potential for application in image processing and in-memory logic operations. First of all, this paper summarizes the material systems, device structure, and physical mechanisms relevant to Hf-FEs devices. Then, for the purpose of achieving efficient neuromorphic computing, the evaluation parameters related to Hf-FEs devices, specifically concerning storage performance and synaptic plasticity, are discussed. Furthermore, the progress of Hf-FEs devices in arrays and hardware integration is systematically reviewed, offering insights for future applications. Finally, this study explores in depth the prospects and challenges of these devices in advanced applications, providing valuable guidance for the development of high-performance neuromorphic computing devices.

## Highlights

- Comprehensively review the material systems, device physics, and performance metrics of complementary metal-oxide-semiconductor-compatible hafnium-based ferroelectric (Hf-FEs), highlighting their potential for next-generation non-volatile memory.
- The review summarized the synaptic plasticity and neuromorphic computing functions implemented by Hf-FEs, emphasizing their advantages in parallelism, energy efficiency, and system-level integration.
- Valuable insights into the development of Hf-FEs toward advanced applications in post-Moore electronics.

## Graphical abstract

**Figure:**
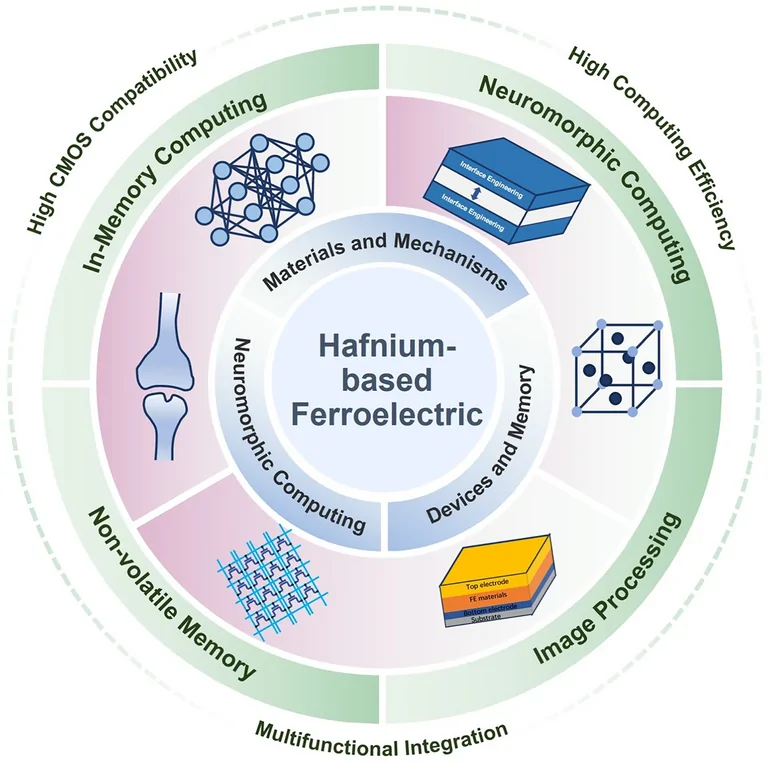

## Introduction

With the exponential growth of the Internet of Things, Cloud Computing, and Artificial Intelligence (AI), the demand for high-performance, low-power computing systems has surged. The physical separation of memory and processing units in the traditional von Neumann architecture leads to frequent data movement, which results in significant energy consumption and severely limits system throughput, forming the so-called "memory wall" bottleneck [10–13]. This efficiency bottleneck has prompted researchers worldwide to actively seek new computing paradigms [10, 14–16], leading to the emergence of neuromorphic computing. These systems, inspired by the brain’s neural structure and operational mechanisms, perform computational tasks in a highly parallel and event-driven manner and are expected to improve energy efficiency by several orders of magnitude. The core of this paradigm shift lies in the ability of non-volatile memory technology to mimic synaptic plasticity, which is the biological basis of learning and memory [17–20]. Traditional perovskite ferroelectric materials, such as Pb(Zr, Ti)O_3_, SrBi_2_Ta_2_O_9_, and BaTiO_3_, have long been extensively studied for their excellent ferroelectric performance. However, their compatibility with silicon-based complementary metal–oxide–semiconductor (CMOS) processes, structural stability under high-temperature annealing conditions, and the interface dead layer and depolarization effects that arise when the thickness is reduced to below 100 nm pose significant limitations. These limitations make it difficult for them to meet the integration size and reliability requirements of advanced microelectronic nodes [21–23]. Concurrently, 2D semiconductors such as MoS_2_ have garnered significant attention due to their atomic thinness, superior electrostatic control, and inherent compatibility with CMOS processing. These properties offer a compelling platform for constructing high-performance, reconfigurable logic and memory devices. Through engineered van der Waals heterostructures, diverse physical mechanisms, including charge trapping and ferroelectric polarization, can be precisely modulated, thereby enriching the device toolkit for implementing versatile in-memory computing and neuromorphic hardware. On the other hand, efforts are also ongoing to find new bulk ferroelectric materials that are inherently compatible with CMOS processes, in order to balance integrability and performance stability [24–26].

**Fig. 1:**
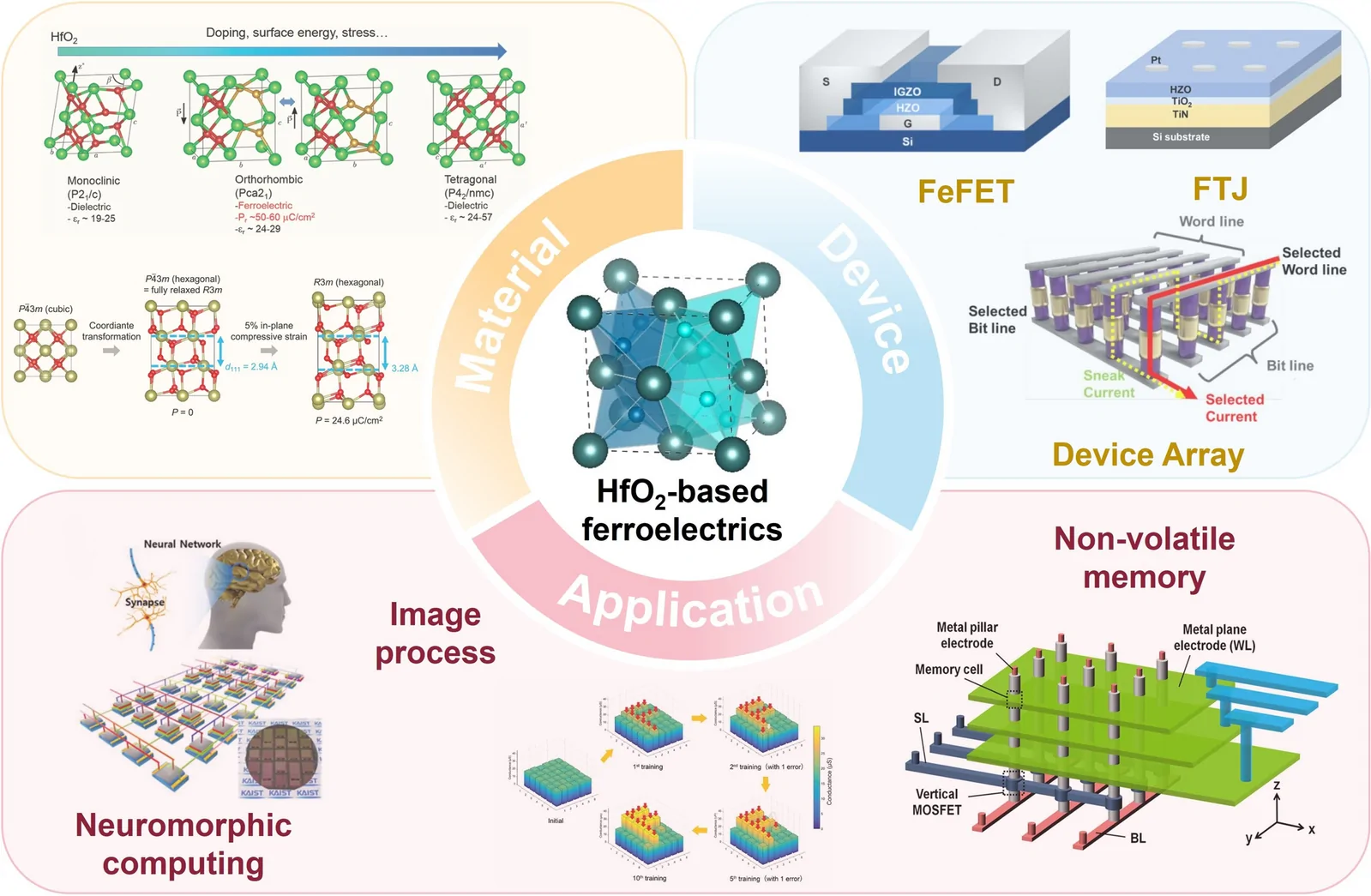
Hf-FEs: Materials, Device Structures, and Applications. HfO_2_-based ferroelectrics Reproduced with permission [1]. Copyright 2022, Springer Nature. Materials Reproduced with permission [2]. Copyright 2017, Royal Society of Chemistry. Reproduced with permission [3]. Copyright 2024, Springer Nature. FeFET Reproduced with permission [4]. Copyright 2022, American Chemical Society. FTJ Reproduced with permission [5]. Copyright 2025, American Chemical Society. Device Array Reproduced with permission [6]. Copyright 2021, American Chemical Society. Neuromorphic computing Reproduced with permission [7]. Copyright 2023, American Chemical Society. Image process Reproduced with permission [8]. Copyright 2018, Royal Society of Chemistry. Non-volatile memory Reproduced with permission [9]. Copyright 2013, American Chemical Society

**Fig. 2:**
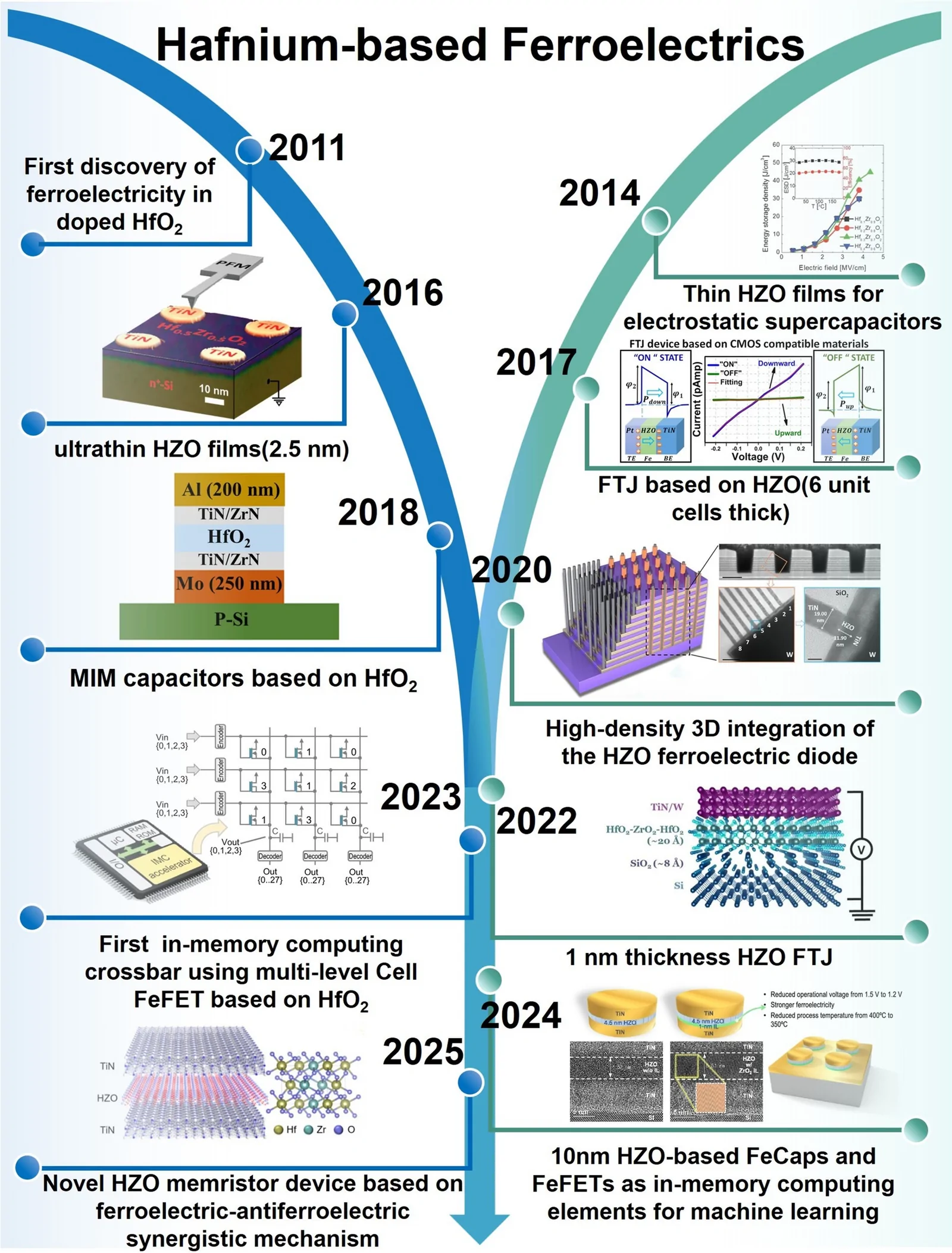
Hafnium—based Ferroelectrics: 2011–2025 Key Advancements. 2014 Reproduced with permission [27]. Copyright 2014, Wiley–VCH. 2016 Reproduced with permission [28]. Copyright 2016, American Chemical Society. 2017 Reproduced with permission [29]. Copyright 2017, American Chemical Society. 2018 Reproduced with permission [30]. Copyright 2018, Elsevier. 2020 Reproduced with permission [31]. Copyright 2020, Springer Nature. 2022 Reproduced with permission [1]. Copyright 2022, Springer Nature. 2023 Reproduced with permission [32]. Copyright 2023, Springer Nature. 2024 Reproduced with permission [33]. Copyright 2024, Springer Nature. 2025 Reproduced with permission [34]. Copyright 2025, American Chemical Society

It is against this backdrop of exploration that a turning point occurred in 2011 with the first discovery of stable ferroelectricity in doped hafnium oxide (HfO_2_) systems [35]. This discovery initiated a surge of research on Hf-FEs (Fig. 1). Unlike perovskite materials, Hf-FEs exhibit several fundamental advantages, including the retention of ferroelectricity at atomic-level thicknesses, natural compatibility with existing CMOS processes, and good tolerance to hydrogen atmosphere annealing treatment, an essential process in the back-end-of-line (BEOL) [36, 37]. These properties originate from the metastable polar orthorhombic phase, which can be stabilized through doping, strain engineering, and surface energy modulation [38–41]. Leveraging the exceptional performance of Hf-FEs devices, device level innovations have advanced rapidly. Therefore, ferroelectric field-effect transistors (FeFETs), ferroelectric tunnel junctions (FTJs), ferroelectric diodes (Fe-Diodes), and three-dimensional ferroelectric random access memory (FeRAM) arrays based on Hf-FEs have been successfully developed. Hf-FEs demonstrate exceptional performance in image processing, logic operations, and hardware integration, with this rapid progress laying a solid technical foundation for achieving three-dimensional monolithic integration of memory, logic, and neuromorphic functionalities, and the field is developing rapidly (Fig. 2) [42–47].

Building upon these excellent achievements, this review is structured as follows to provide a comprehensive perspective on Hf-FEs technology. Section 2 begins with an in-depth examination of Hf-FEs materials and devices, covering the material systems, device structures, underlying physical mechanisms, and key performance metrics. We then discuss their primary applications in memory technology, setting the stage for their computational use. Section 3 is dedicated to neuromorphic computing based on Hf-FEs. It first explores the emulation of synaptic plasticity using these devices, then reviews progress in neuromorphic system implementation, and finally details their applications in image processing and in-memory logic operations. Section 4 critically assesses the major challenges and prospects facing the field, outlining pathways for future development. Finally, Section 5 provides a concise conclusion, summarizing the transformative potential of Hf-FEs in advancing post-Moore electronics. This structure aims to guide the reader from fundamental principles to advanced applications and future outlooks, systematically illuminating the role of Hf-FEs in next-generation computing.

## Hafnium-Based Ferroelectrics

### Material System

**Fig. 3:**
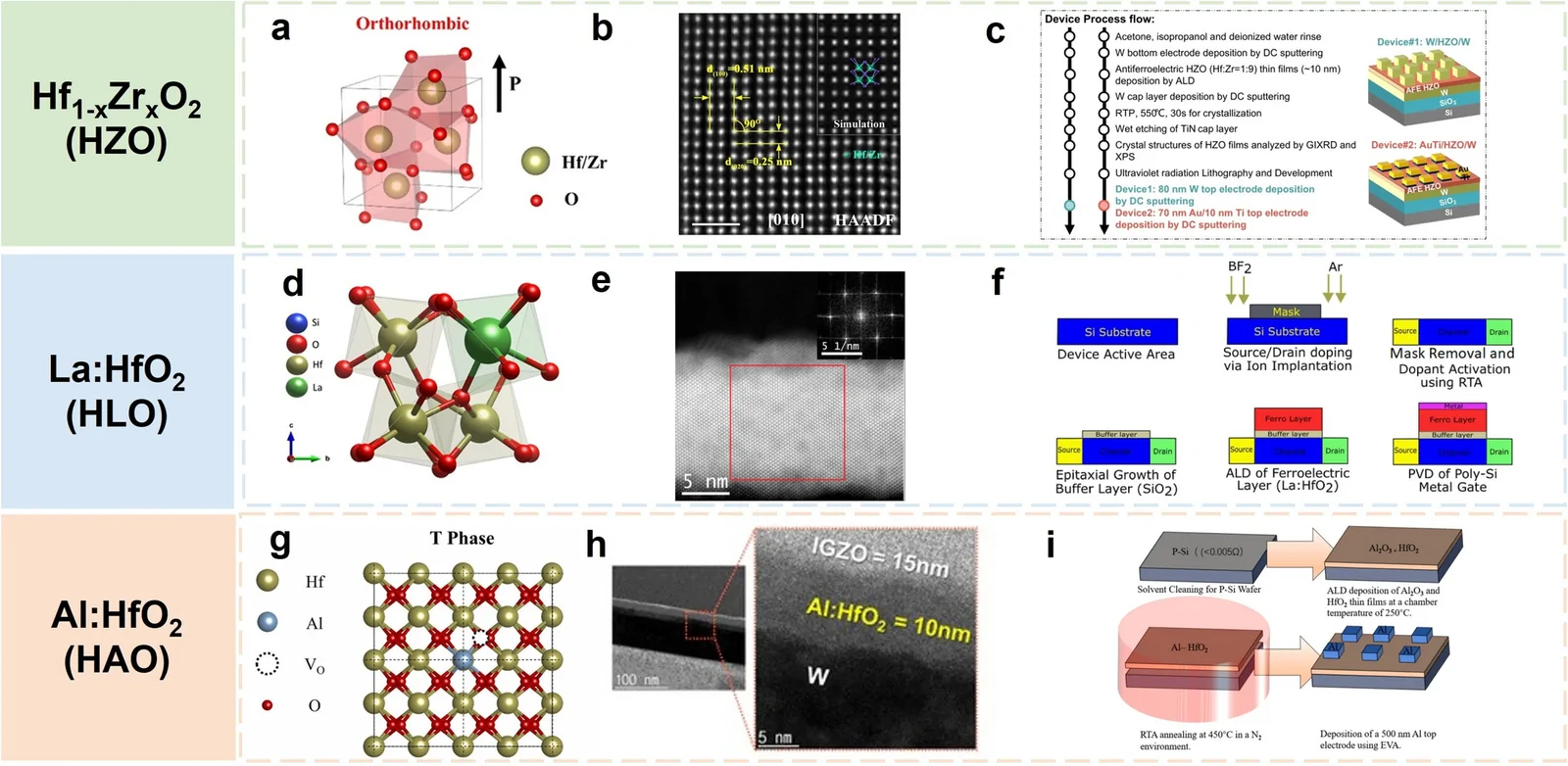
Hafnium-based materials and fabrication diagram of hafnium-based devices. **a** Crystal structure diagram of o-phase in Zr-doped hafnium oxide [57]. Copyright 2023, American Chemical Society. **b** STEM-HAADF image of the HZO [31]. Copyright 2020, Springer Nature. **c** Process flow of AFE HZO-based devices [58]. Copyright 2024, American Chemical Society. **d** La-doped HfO_2_ structure diagram [59]. Copyright 2020, American Chemical Society. **e** TEM images of orthorhombic HfO_2_ of the 10 cat% La with [101]-zone axis [55]. Copyright 2019, American Chemical Society. **f** Process flow of La:HfO_2_ FeTFET [60]. Copyright 2024, Elsevier. **g** Crystal structures of t-phase HfAlO_x_ Fig. 3 Copyright 2024, Elsevier. **h** TEM image of Al:HfO_2_ thin film [61]. Copyright 2021, American Chemical Society. **i** Process flow of HfAlO device [62] . Copyright 2025, Elsevier

In hafnium-based materials, HfO_2_-based materials are known for their stable and controllable properties. The ferroelectricity of these materials originates from the formation of asymmetric centers within the metastable polar orthorhombic phase (o-phase, Pca2_1_), a process induced by mechanical constraints [35]. Unlike traditional perovskite materials, HfO_2_-based materials are highly compatible with mainstream CMOS-integrated circuit technology. This compatibility greatly enhances their application prospects in the semiconductor industry [48–50]. However, given the metastable nature of the ferroelectric orthorhombic phase in HfO_2_, suppressing the formation of non-ferroelectric phases requires careful control of various process parameters. Chemical doping, by altering the local bonding environment within the HfO_2_ film and inducing strain, offers a viable approach to stabilize the ferroelectric orthorhombic phase [3, 51, 52]. Beyond the initially reported silicon (Si) doping, numerous other elements including zirconium (Zr), lanthanum (La), aluminum (Al), and yttrium (Y) have been employed to optimize the ferroelectric properties of HfO_2_ [37, 53–56]. Figure 3 shows some common doped HfO_2_ ferroelectric materials.

Among various dopants, the Zr dopant is the most widely used in HfO_2_ due to the very similar physical and chemical properties of Zr and Hf. Fig. 3a shows the crystal structure of the orthorhombic phase in Zr-doped HfO_2_, which is the key basis for its ferroelectric performance. The microstructure characteristics of the HZO thin film can be clearly observed in Fig. 3b through STEM-HAADF of HZO. For Zr-doped HfO_2_, the dopant concentration can be stably maintained at 50% when the maximum ferroelectric polarization is achieved, facilitating uniformity and reproducibility in mass production [53, 64]. In addition, HZO thin film can be crystallized within a wider and lower temperature range (400–600 °C) compared to other dopant-doped thin films, which is beneficial to BEOL integration [65]. Fig. 3c shows a device fabrication flow chart based on anti-ferroelectric HZO.

Lanthanide elements are also considered promising candidate elements for doping HfO_2_ [55, 65]. The crystal structure diagram of La-doped HfO_2_, intuitively reflecting the doping state of La atoms in the HfO_2_ lattice, is presented in Fig. 3d. And Fig. 3e shows the TEM image of La:HfO_2_ thin film, revealing the crystal structure details and microscopic morphology of the La:HfO_2_ thin film. Given the relatively large ionic radius and low electronegativity of La atoms, Jeon et al. demonstrated that La doping in hafnium-based FETs can reduce oxygen vacancy concentration in HfO_2_ thin films, minimizing defects and improving gate bias stability [66]. Among various dopants, La exhibits the strongest stabilizing effect on HfO_2_ ferroelectrics, which is beneficial for achieving excellent ferroelectric performance. For example, the La:HfO_2_ thin films reported by U. Schroeder et al. showed a *2P*_*r*_ of 55 μC cm^−2^ after annealing at 800 °C [55]. Fig. 3f shows the process flow of the La:HfO_2_ FeTFET, further illustrating the application of La-doped HfO_2_ materials in low-power FeTFET design.

Besides Zr and La, some elements commonly used in the semiconductor field, such as Si and Al, are also frequently used as dopants [67, 68]. Figure 3g shows the crystal structure of HfAlO_x_, revealing the structural characteristics of HfO_2_ lattice formation resulting from Al doping. The extremely small ionic radius of Al causes Al doping to introduce compressive stress lattice strain, which suppresses the formation of the monoclinic phase during the rapid thermal annealing process. This strain-dominated stabilization mechanism enables strong ferroelectricity in ultra-thin films while maintaining a considerable remnant polarization [68, 69]. Figure 3h presents a TEM image of the Al:HfO_2_ thin film, allowing for further observation of its microstructure and thickness characteristics. Figure 3i illustrates a fabrication process for HfAlO devices, offering specific process guidance for device applications of Al-doped HfO_2_ materials.

In summary, the performance of hafnium-based ferroelectric materials is highly dependent on the selection of doping elements, with different dopants being adapted to differentiated application scenarios by modulating lattice strain, oxygen vacancy concentration, and phase stability. The HZO system formed by Zr doping has become a mainstream choice for high-density FeFET and FeRAM arrays because of its high remanent polarization and good CMOS process compatibility, but its high coercive field may lead to an increase in operating voltage [58]. La doping can significantly suppress oxygen vacancies and improve high-temperature stability, maintaining strong ferroelectricity even after annealing at 800 °C, which is suitable for high-reliability storage and logic devices requiring back-end high-temperature integration [55]. Although Si doping first revealed the ferroelectricity of HfO_2_ and has a mature process, its poor endurance means that it is mostly used for basic research and prototype verification. Compressive strain introduced by Al doping is conducive to stabilizing the ferroelectric phase in ultra-thin films, which demonstrates potential in ultra-thin FTJs and three-dimensional stacked neuromorphic devices [61]. Consequently, doping engineering has become a critical method for customizing the ferroelectric performance of HfO_2_, meeting diverse application needs ranging from high-density memory to low-power neuromorphic computing.

Co-doping strategies have achieved breakthroughs in the overall performance of HfO_2_-based ferroelectric materials through synergistic effects between different elements. The core synergistic mechanisms include three key aspects:

1. Synergistic regulation of oxygen vacancies: For example, in La–Al co-doping, Al promotes the generation of oxygen vacancies to stabilize the ferroelectric phase, while La suppresses their excessive migration. This synergy enhances polarization strength and ensures high endurance, as evidenced in a 3D ferroelectric memory based on La–Al co-doped HfO_2_ thin films, which exhibits excellent ferroelectric properties and outstanding memory performance [70] .
2. Lattice strain synergistic equilibrium: This can be achieved by optimizing the concentration and spatial distribution of dopants. For instance, tuning process parameters to induce favorable in-plane shear stress promotes the formation of the ferroelectric orthorhombic phase. The study by Liu et al. demonstrates that when Al-doped HZO thin films are prepared with a cycle ratio of 1:48, the ferroelectric performance is significantly improved [71] .
3. Synergistic interface engineering: As shown by Mehrdad Ghiasabadi Farahani et al., constructing multilayer structures by inserting La-doped HfO_2_ subnanometer layers is an effective strategy for enhancing the performance of HZO-based ferroelectric films. This approach is particularly suitable for high-performance electronic devices such as non-volatile memory, ferroelectric memory, and capacitive memory elements [72] .

In conclusion, the ferroelectric performance of HfO_2_ can be precisely controlled through doping strategies, and the effects of different dopants, concentrations, and thin film thicknesses on key parameters such as remanent polarization, coercive field, and thermal stability are complex and dispersed across various research reports. To provide an intuitive comparison and trend summary, we have summarized the performance parameters of some doped HfO_2_ thin films in Table 1.

**Table 1:** Performance comparison of different doped HfO_2_ ferroelectric thin films

Dopants	Doping concentration [mol%]	Film thicknesses [nm]	Stack configuration	2*P*_*r*_ [µC cm^−2^]	*E*_*c*_ [MV cm^−1^]	Thermal stability	References
Zr	50	4	TiN/HZO/TiN	15	N/A	Retention > 10^4^ s @ 85 °C	[73]
Zr	50	8.9	W/HZO/W	53.9	1.37	Excellent thermal stability	[74]
Zr	50	10	Au/HZO/LSMO	40	2.5	Excellent thermal stability with no significant wake-up effect	[75]
Zr	50	15	W/HZO/W	18.3	1.4	Retains data at 250 °C	[76]
Si	4.2	10	Pt/HSO/TiN	15	1.0	Excellent thermal stability	[77]
Si	4.6	10	TiN/HSO/TiN	16–34	N/A	Saturated polarization state exhibited virtually no degradation after 1000 h of baking at 125 °C	[78]
La	2	8.5	Pt/HL/LSMO	44	4.42	10 years retention at 85 °C	[79]
La	3	10	W/IGZO/HLO/TaN	16.8	2.2–3.0	Good thermal stability @ 700 °C, 10-s RTA annealing	[66]
Al	3	4.5	W/HAO/W	20	8.6	Excellent ferroelectric performance after annealing at 850 °C	[61]
Al	4.8	16	TiN/HAO/TiN	10	1.0	Ferroelectricity can still be formed and enhanced at annealing temperatures up to 1000 °C	[54]
La + Al	Al:4.2 La:2.17	10	W/HfAlLaO/W	22	1.6	10^8^ cycles at 85 °C	[70]

### Device Structure

HfO_2_ possesses unique properties, most notably its CMOS compatibility and scalability, which enable its integration into device architectures for various memory and logic applications. In this section, several structures of Hf-FEs devices will be explored, with a focus on their working principles, advantages, and scaling potential.

#### FeFETs

FeFETs, by directly embedding non-volatile memory functions within transistors, enable novel memory computing, neuromorphic computing, and other computing paradigms. Structurally, they are compatible and scalable with CMOS technology. FeFETs switch ferroelectric polarization states via field control. This polarization reversal alters the transistor’s threshold voltage, which is then used to represent "0" or "1" states; this polarization state is maintained even after power is turned off. Compared to perovskite-structured ferroelectric materials, hafnium-based FeFETs exhibit higher coercive fields and greater resistance to the depolarization field, resulting in superior retention characteristics [91, 92].

**Fig. 4:**
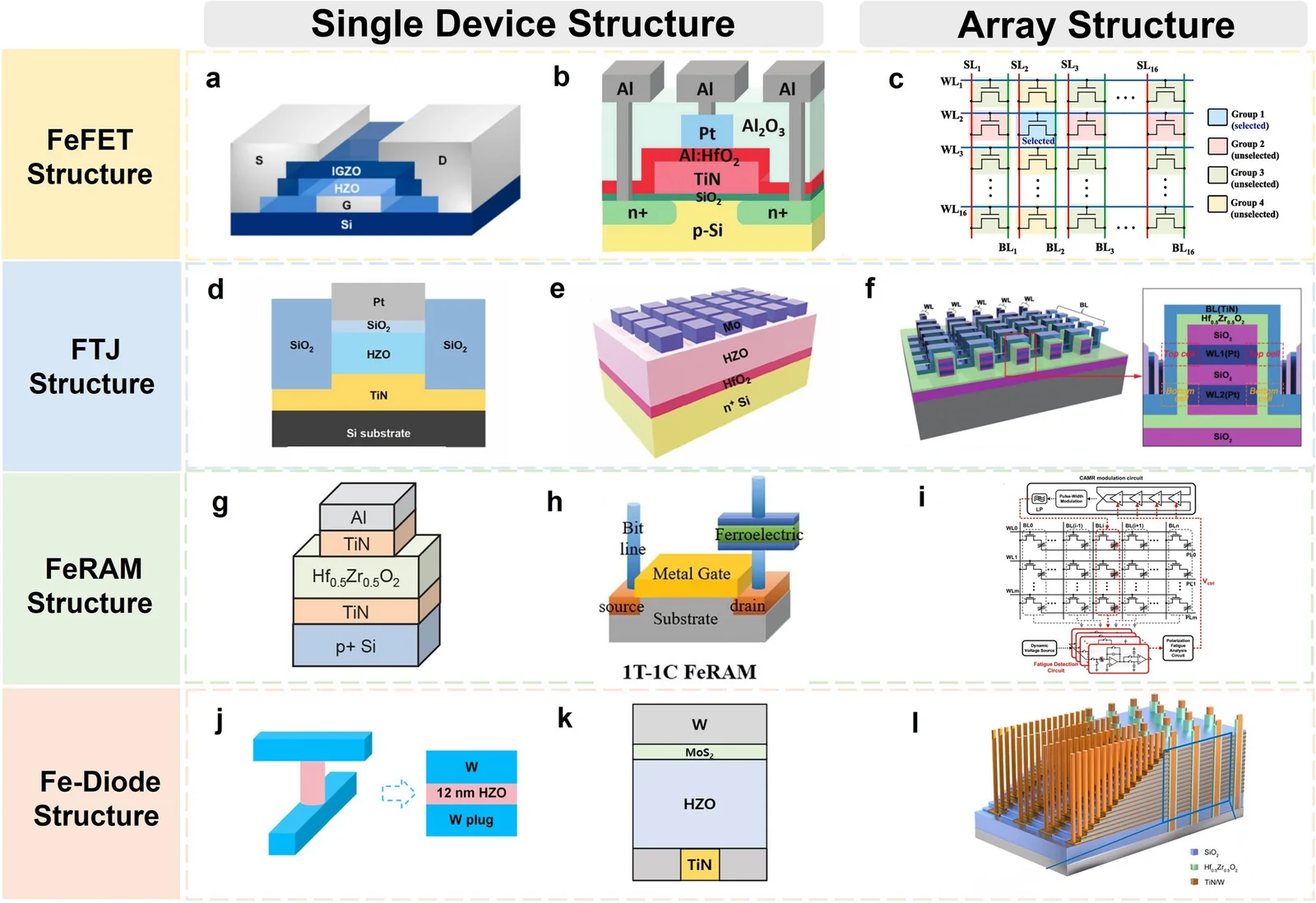
Structure of hafnium-based ferroelectric devices. **a** Schematic of the MFS FeFET device structure [4]. Copyright 2022, American Chemical Society. **b** Schematic of the MFMIS FeFET structure [80]. Copyright 2020, Royal Society of Chemistry. **c** Circuit diagram of a 16 × 16 FeFET AND array [81]. Copyright 2024, Elsevier. **d** Schematic diagram of the device structure of a Pt/SiO_2_/HZO/TiN FTJ [82]. Copyright 2024, Wiley–VCH. **e** Schematic diagrams of FTJs with MFIS structures [83]. Copyright 2024, Wiley–VCH. **f** Schematic diagram of high-density 3D vertical HZO-based FTJS array [8]. Copyright 2018, Royal Society of Chemistry. **g** Schematic structure of ferroelectric capacitors [84]. Copyright 2022, American Chemical Society. **h** Schematic diagram of 1 T-1C FRAM structure [85]. Copyright 2022, Wiley–VCH. **i** Circuit architecture of FeCAP-based memory array and CAMR recovery circuit [86]. Copyright 2025, American Chemical Society. **j** Diagram of Fe-Diodes with a W/HZO/W plug structure [87] . Copyright 2025, AIP Publishing. **k** Diagram of Fe-diodes based on a W/MoS_2_/HZO/TiN stack [88]. Copyright 2025, Wiley–VCH. **l** Structure of the 16-layer 3D Fe-diode [90]. Copyright 2025, Springer Nature

Fig. 4a illustrates an FeFET employing an MFS stacked structure. The HZO/IGZO-based FeFET exhibits excellent electrical performance, featuring not only a large memory window of 2 V and a near-ideal subthreshold swing of 63 mV dec^−1^, but also achieving a high on/off current ratio of 3.5 × 10^6^ and an ultra-low off-state leakage current down to the pA μm^−1^ level [4].

Building upon this, Fig. 4b demonstrates an MFMIS-stacked FeFET. This structure successfully mimics biological synaptic function, enabling electrical modulation of synaptic weights and low-voltage operation, which makes it suitable for low-power neuromorphic computing. In addition, integrating such devices into 3 × 3 synaptic arrays effectively verifies the electrically modifiable weighted-sum operation, providing a foundation for the future implementation of neuromorphic computing in AI hardware [80] . The integrated application of FeFET in array architectures has been actively explored by researchers, exemplified by the 16 × 16 FeFET AND array shown in Fig. 4c. This array achieves selective write operations and incorporates a biasing scheme for write inhibition, which is realized solely through bit line voltage. These capabilities highlight the potential of FeFET in high-density, low-power neuromorphic memory applications [81].

Despite the significant progress made in FeFET technology over the past decade, its development remains limited by several key challenges, including unstable memory window behavior, significant device-to-device variation, and the detrimental "wake-up"/"fatigue" effects observed in the ferroelectric layer. Furthermore, scaling effects impose significant limitations when FeFETs are integrated at high density. To address these challenges, future research can be conducted in several directions. These include exploring novel ferroelectric materials and superior interface layers through materials and interface engineering, as well as developing novel device structures such as three-dimensional stacking and multi-gate structures through structural innovation and optimization. Through interdisciplinary collaborative innovation, FeFET technology is expected to overcome current bottlenecks and play a key role in future high-energy-efficiency computing.

#### FTJs

Unlike three terminal FeFETs, the FTJs are two terminal devices. Its basic structure comprises two conductive electrodes separated by a nanometer-thick ferroelectric layer, which serves as an energy barrier. This concept was initially proposed by L. Esaki et al. in 1971 and termed "polarity switch" [93]. However, it was not until 2012 that a functional solid-state device utilizing the FTJ principle was demonstrated [94]. In FTJs, charge carriers are transported via quantum mechanical tunneling through the ultra-thin ferroelectric barrier. The polarization state of the ferroelectric material governs the tunneling resistance, leading to two distinct resistance states, which enable the storage of binary or higher order information.

FTJs are considered promising candidates for constructing artificial synapses and high-density, ultrafast memories. As shown in Fig. 4d, a Pt/SiO₂/HZO/TiN FTJ device achieves sub-nanosecond switching speed of 500 ps at voltages ≤ 5 V. This FTJ exhibits a low write current density of 1.3 × 10^4^ A cm^−2^, endurance of over 10^7^ cycles under 85 °C, and a large read current density of 88 A cm^−2^ at voltages below 0.1 V, thus demonstrating its suitability for future FTJ-based neural network computing [82] . Fig. 4e illustrates an FTJ employing an MFIS structure with strong ferroelectric properties. Interestingly, the authors found the MFIS structure to be superior to the MIFS structure with respect to ferroelectric properties, exhibiting a higher remnant polarization of approximately 69 μC cm^−2^. Furthermore, the MFIS FTJ exhibited reduced leakage current, a higher tunneling electroresistance ratio, and a thinner interfacial dead layer during short-pulse switching, thus providing a promising pathway for future development [83]. Figure 4f illustrates a 3D vertically integrated ferroelectric HZO-based FTJ array designed to emulate the full functionality of biological synapses. The fabricated 3D vertical FTJ synapse features high integration density and superior performance metrics, including analog conductance modulation under training schemes, low-energy consumption per synaptic weight update (1.8 pJ spike^−1^), and excellent repeatability (> 10^3^ cycles) [8].

FTJs hold great promise for ultra-high-density non-volatile memory applications. However, current FTJ devices based on HfO_2_ also face some challenges: the underlying tunneling mechanism remains unclear, and a further improvement in the switching ratio is needed to achieve reliable sensing operations. It is expected that the ferroelectric domain switching process can be directly observed at the atomic scale in the future by utilizing in situ electrical testing and advanced microscopic characterization techniques, which will reveal its dominant tunneling mechanism. The intrinsic polarization strength of HfO_2_ ferroelectric thin films can be precisely controlled through material and band engineering to obtain more stable and superior ferroelectric performance, and to explore novel device architectures. Collaborative innovations in physical mechanisms, material systems, and device structures will pave the way for the next generation of ultra-high-density memory and neuromorphic computing applications.

#### FeRAMs

The basic concept of FeRAM can be traced back to the 1950s. In this memory architecture, the two terminals of a ferroelectric capacitor (FeCAP) are connected to lines in the vertical direction, forming memory arrays. The one transistor-one capacitor (1T-1C) configuration is the most common FeRAM implementation. In a 1T-1C FeRAM cell, the memory cell is composed of an access transistor integrated with a FeCAP. Binary information is stored via the capacitor’s switchable polarization states (up or down), and the access transistor enables random access read and write operations [95–97].

As shown in Fig. 4g, the core structure of a FeCAP consists of a ferroelectric material thin film sandwiched between top and bottom electrodes. Hafnium-based materials exhibit distinct ferroelectric hysteresis loop characteristics, maintaining two stable polarization states and thereby enabling data storage. Embedded ferroelectric memory was successfully realized by Kasidit et al. using an HZO-based FeCAP with its thickness reduced to 4 nm. Test results indicate that the device exhibits excellent reliability even at a low operating voltage of 1.2 V; it achieves an endurance of at least 10^12^ cycles at a 200 kHz operating frequency, coupled with a 10 year data retention capability [84] . This achievement demonstrates that reducing the thickness of the HZO thin film effectively lowers the operating voltage and improves endurance. This provides a crucial pathway for developing low-power, high-reliability back-end compatible embedded FeRAM.

A single transistor combined with a FeCAP constitutes the most common 1T-1C cell structure in FeRAM. As shown in Fig. 4h, within this cell, the gate of the access transistor is connected to the word line for gating control, the source is connected to the bit line to facilitate data reading and writing, and the other terminal of the FeCAP is connected to the plate line to apply the necessary voltage pulses during operation.

In practical applications, millions of 1T-1C cells are integrated and organized into high-density memory arrays. Figure 4i shows a FeCAP-based memory array architecture. To mitigate ferroelectric fatigue in HZO arrays, Huang et al. introduced a design scheme integrating a CAMR recovery circuit, which enables dynamic detection of fatigue states and signal recovery. This technology, through its built-in detection and recovery mechanisms, significantly enhances FeRAM’s reliability and lifetime, offering a practical solution for its application as an on-chip storage element and in next-generation non-volatile memory and "More than Moore" devices, and thereby demonstrating promising development prospects [86] .

#### Fe-Diodes

Besides three terminal transistors and two terminal junction devices, Fe-diodes, owing to their simple structure and ultra-high scalability, have emerged as promising storage elements, especially suitable for ultra-high-density cross-array integration. Unlike FTJs that rely on tunneling resistance, their working principle is based on Schottky barrier modulation regulated by polarization reversal, with MFS or MFM structures. The ferroelectric polarization direction can realize current rectification, forming a significant non-volatile on/off ratio. This inherent self-selection characteristic can effectively suppress sneak currents in cross arrays, eliminating the need for an additional selector, greatly simplifying circuit design and improving array density [31, 98, 99].

Figure 4j shows a Fe-diode fabricated with a W/HZO/W plug structure. The device exhibits stable polarization characteristics and a good memory window, enabling nondestructive readout at low bias voltages. Based on the switchable bidirectional rectification characteristics of this device, Han et al. constructed a 2-bit multiplication scheme that requires only 11 devices and 16 steps to obtain a 4-bit product output, with a power consumption of approximately 11 fJ throughout the entire operation. This demonstrates the feasibility of applying Fe-diodes to self-selective high-density crossbar arrays [87].

Fe-diodes operating through polarization-driven Schottky barrier modulation often exhibit high leakage currents and unstable switching behavior because of insufficient barrier control and interface defects. By introducing a buffer layer at the two-dimensional MoS_2_ top electrode/FE interface (Fig. 4k) and increasing the HZO thickness from 3 to 8 nm, Hwang et al. shifted the dominant conduction mechanism from direct tunneling to Schottky emission. This resulted in more robust polarization-induced barrier modulation, a significantly improved on/off ratio, a high current density of up to 50 A cm^−2^ (read at 3 V), endurance exceeding 10^10^ cycles, and stable memory retention for 10 years at room temperature. This confirms the exceptional scalability and strong potential of this Fe-diode design in next-generation integrated memory applications [90].

Beyond achieving performance breakthroughs at the individual device level, the potential for applying Fe-diodes in arrays has also been initially validated, paving the way for their use in high-density integrated memory and computing. Huang et al. successfully fabricated a 16-layer stacked Fe-diode array (Fig. 4l). The core innovation lies in unifying the storage and random source generation functions in the same three-dimensional arrays. This "memory-computing-random integrated" architecture achieves an area efficiency of up to 0.06 F^2^ state^−1^ and a low programming energy consumption of 25 fJ. More importantly, the inherent rectification characteristics and three-dimensional stacking capability of Fe-diodes provide an ideal device solution for constructing high-density, self-selected, low-crosstalk crossbar arrays. This work demonstrates, from the perspective of array scale, the great potential of Fe-diodes in realizing the next generation of high-energy efficiency and high-area efficiency edge intelligent computing hardware [90].

Recent advances in Fe-diodes based on HfO_2_ have demonstrated remarkable performance, including nanosecond switching speeds, excellent endurance, and multilevel operation capabilities. However, challenges remain in achieving high uniformity, reliable polarization reversal at low voltages, and complementary diode pairing for practical memory logic applications.

As previously mentioned, Hf-FEs devices exhibit superior performance in terms of switching speed, endurance, and power consumption, making them central to building non-volatile storage and neuromorphic computing hardware. To systematically evaluate the comprehensive performance and applicable scenarios of these device architectures, Table 2 provides a cross-benchmark comparison of their key metrics.

**Table 2:** Comparison of different device performances based on HfO_2_ ferroelectric materials

Device type	Structure	FE material	Switching speed	Retention	Endurance	Power consumption	References
FeFET	MFS	HZO (10 nm)	N/A	> 10^4^ s	> 10^6^ cycles	pA/μm level off-state leakage current	[4]
FeFET	MFS	HZO (10 nm)	40 ns	> 1500 s	8 × 10^6^ cycles	Extremely low writing energy	[92]
FeFET	MFIS	HZO (5 nm)	1 μs	> 10^5^ s	> 10^5^ cycles	Low operating voltage of 3.8 V	[99]
FeFET	MFIS	HSO (10 nm)	300 ns	N/A	> 10^5^ cycles	Low gate leakage current	[100]
FeFET	MFMIS	HAO (10 nm)	500 ns-10 μs	> 10^4^ s	N/A	2 V to 5 V low operating voltage	[80]
FTJ	Pt/SiO/HZO/TiN	HZO (2 nm)	500 ps	> 10^5^ s	> 10^7^ cycles	0.12 fJ/bit write energy	[82]
FTJ	Pt/HZO/LSMO	HZO (2 nm)	< 500 ns	> 10^5^ s	N/A	N/A	[101]
FTJ	Pt/HZO/TiO_2_/TiN	HZO (4.2 nm)	50 ns	> 10^5^ s	> 2 × 10^8^ cycles	< 10 fJ	[5]
FTJ	TiN/HZO/TaN/W	HZO (5.5 nm)	N/A	> 10 years	10^8^ cycles	Low operating voltage from 1.5 V to 3.5 V	[101]
FTJ	TiN/HZO/Pt	HZO (9 nm)	100 ns	N/A	> 10^3^ cycles	1.8 pJ/spike	[8]
FeRAM	TiN/HZO/TiN	HZO (4 nm)	N/A	> 10 years	> 10^12^ cycles	Low operating voltage 1.2 V	[84]
FeRAM	TiN/HZO/TiN	HZO (8 nm)	N/A	> 10 years	> 10^9^ cycles	Low operating voltage 2.0 V	[95]
FeRAM	TiN/HZO/TiN	HZO (10 nm)	N/A	> 10 years	> 10^13^ cycles	Low operating voltage 1.5 V	[86]
Fe-Diode	MOFM	HZO (7 nm)	800 ps	> 10 years	> 10^9^ cycles	0.8 fJ	[102]
Fe-Diode	W/MoS_2_/HZO/TiN	HZO (8 nm)	200 ns	> 10 years	> 10^10^ cycles	158.5 fJ (per operation)	[88]
Fe-Diode	TiN/HZO/TiN	HZO (10 nm)	20 ns	> 5 × 10^4^ s	> 10^9^ cycles	Low operating current of 1 μA	[31]

### Physical Mechanism

**Fig. 5:**
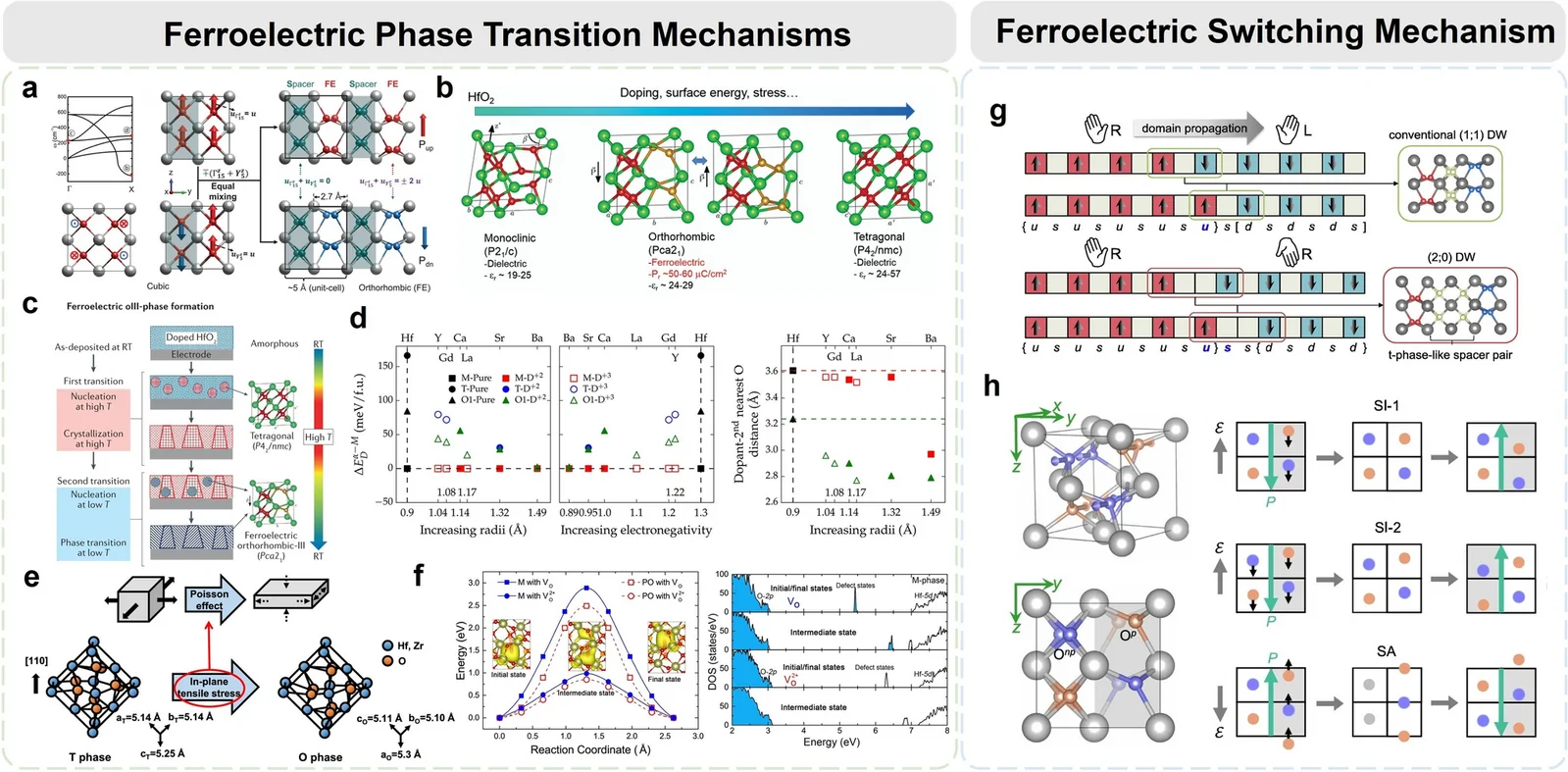
Physical Mechanism. **a** Structural origin of alternating ferroelectric and non-polar layers in orthorhombic HfO_2_ [103]. Copyright 2020, American Association for the Advancement of Science. **b** Schematic diagram for the evolution of phases in doped HfO_2_ films [2]. Copyright 2017, Royal Society of Chemistry. **c** Schematic diagram for the formation of the ferroelectric orthorhombic phase in doped HfO_2_ thin films during a rapid thermal process [51]. Copyright 2022, Springer Nature. **d** Chemical trends of relative energies among M, T, and P-O1 phases of hafnium with different dopants [104]. Copyright 2017, American Chemical Society. **e** Phase transition of HZO under stress [4]. Copyright 2022, American Chemical Society. **f** Ferroelectric structural transition of hafnium oxide induced by charged oxygen vacancies [105]. Copyright 2021, American Physical Society. **g** Schematic illustration of the DW motions [106]. Copyright 2021, Elsevier. **h** Polarization switching pathways in ferroelectric HfO_2_ [107]. Copyright 2023, American Physical Society

HfO_2_ exhibits complex phenomena with the coexistence of multiple phases, and the origin of ferroelectricity in ferroelectric hafnia remains controversial. Using first-principles computing, Lee et al. discovered that flat bands of polar phonons, and the resulting local dipoles, induce scale-free ferroelectric order in HfO_2_. Therefore, the emergence of these flat phonon bands in HfO_2_ provides an explanation for the origin of its scale-free ferroelectricity (Fig. 5a) [103].

Under ambient conditions, the monoclinic phase (m-phase, P2_1_/c) is the most stable phase of HfO_2_. As the temperature increases above 1973 K, the stable phase transforms into the tetragonal phase (*t*-phase, P4_2_/nmc), and subsequently into the cubic phase (c-phase, Fm3m) at 2773 K. Additionally, phase transitions can be induced by pressure [51]. It is now generally accepted that ferroelectricity in hafnium-based thin films originates from the o-phase. Among numerous phases, the polar phases of HfO_2_ are metastable compared to the non-polar m-phase. A fundamental challenge in fabricating ferroelectric HfO_2_-based thin films is suppressing the formation of the non-polar monoclinic phase while promoting the emergence of polar phases. Various extrinsic factors, including temperature, oxygen vacancy concentration, dopants, strain, and surface/interface effects, can influence the phase transition (Fig. 5b). Figure 5c illustrates the formation of the ferroelectric orthorhombic phase in doped HfO_2_ thin films during rapid thermal processing.

#### Ferroelectric Phase Transition Mechanisms

The ferroelectricity in HfO_2_ was first discovered in Si:HfO_2_, which immediately aroused great interest in the dopant effect. It is understood that dopants stabilize phases by modulating size and influencing the formation of oxygen vacancies [104, 108]. Rohit Batra et al. employed high-throughput first-principles density functional theory (DFT) computing to investigate the effect of nearly 40 dopants on the phase stability of HfO_2_. They found that dopants with a larger ionic radius and lower electronegativity most effectively stabilize the P-O1 phase. Their study predicts that lanthanide elements, the lower half of the alkaline earth metal series like Ca, Sr, Ba, and Y, are the most favorable dopants for enhancing ferroelectricity in HfO_2_ (Fig. 5d) [104].

Epitaxial strain is also considered a key factor in stabilizing the metastable o-phase in HfO_2_ thin film. It is understood that through in-plane tensile stress, the Poisson effect causes out-of-plane contraction (transverse strain), leading to lattice distortion and driving the *t*-phase to transform into the o-phase (Fig. 5e) [4]. The effect of biaxial strain on the thermodynamic stability of HfO_2_ polymorphs has been extensively studied. Fan et al. demonstrated that tensile strains greater than 3% render the Pca2 phase more stable than the antipolar Pbca phase [109]. Furthermore, strain can synergistically interact with other extrinsic factors such as doping, oxygen vacancy, and interfacial effects to enhance the stability of the ferroelectric phase.

The variation in oxygen vacancy concentration is a crucial factor in the phase evolution of HfO_2_. Numerous studies have demonstrated the significant impact of oxygen vacancies on the ferroelectric performance of HfO_2_-based thin films. Given that oxygen vacancies are intrinsic to HfO_2_, experimental observations indicate that the migration of these vacancies from the interface to the bulk region triggers a transition from the m-phase to the polar o-phase. Critically, a higher concentration of oxygen vacancies suppresses the monoclinic phase while stabilizing the polar o-phase [110, 111]. Through DFT calculations, He et al. demonstrated that when the concentration of doubly charged oxygen vacancies (VO^2+^) exceeds 2%, the ferroelectric polar o-phase becomes thermodynamically more stable than the m-phase. This confirms VO^2^⁺ as a stabilizer of the ferroelectric phase in HfO_2_ (Fig. 5f) [105].

#### Switching Mechanisms

Polarization switching is a fundamental property of ferroelectrics. Understanding the microscopic mechanisms is not only of fundamental scientific importance but also crucial for their application in future electronic devices. Currently, the microscopic mechanisms of polarization switching in fluorite-structured ferroelectrics remain unclear, although several theories, such as domain wall motion and oxygen ion migration, have been proposed.

Microscopic mechanism:

Domain walls (DWs) are interfaces separating domains with distinct polarization orientations. In HfO_2_, polarization switching driven by DW motion primarily occurs via low-energy-barrier (2;0)-type DW pathways. Compared to the high-energy barrier (1.36 eV) of conventional (1;1)-type DWs, the (2;0) pathway exhibits a significantly reduced barrier of only 73 meV due to its geometric similarity to the t-phase. This process involves the movement of a DW from a polarization layer number of *n*_*u*_ = N to *n*_*u*_ = N + 1. The tetragonal transition state can be further stabilized under tensile strain. Applying a 0.4% strain reduces the energy of the pseudo-monoclinic intermediate state, effectively eliminating the transition barrier. Consequently, polarization reversal controlled by electric fields or mechanical stress becomes highly efficient (Fig. 5g) [106].

In Pca2_1_-structured HfO_2_, ferroelectricity originates from the asymmetric displacement of oxygen ions within the unit cell. These oxygen ions are categorized into two types: polar oxygen ions (*Oₚ*), which generate the net polarization along the Z-axis, and non-polar oxygen ions (*Oₙₚ*), located in non-polarizing regions. Polarization reversal involves the concerted migration of oxygen ions, primarily through two distinct pathways: The Intralayer Migration Pathway: *Oₚ* ions displace within their original positions, directly reversing the orientation of the local dipole moment. The Interlayer Migration Pathway: *Oₙₚ* ions migrate across the polar/non-polar boundary, achieving polarization reversal by inverting the X_2_^−^ vibrational mode (represented by the gray-colored transition state). During this process, the identities of *Oₙₚ* and *Oₚ* ions are exchanged, leading to the reversal of the net polarization direction. (Fig. 5h) [107].

Phenomenological models:

The aforementioned atomic-scale images reveal the physical origin of polarization reversal; however, the macroscopic switching dynamics and reliability of Hf-FEs thin films in practical device applications are strongly dependent on their polycrystallinity, defect distribution, phase boundary disorder, and spatial inhomogeneity. To quantify these complex behaviors, a series of phenomenological models has been widely adopted, among which the Kolmogorov–Avrami–Ishibashi (KAI) model and the nucleation-limited switching (NLS) model are critical [112, 113].

Studies have shown that the switching of polycrystalline hafnium-based ferroelectric thin films is mainly dominated by the nucleation process, and domain wall motion is limited. The switching image described by the classical KAI model, which involves free lateral expansion after nucleation, is often not applicable here. The experimentally observed non-exponential relaxation, broadened switching time distribution, and partial polarization switching characteristics are highly consistent with the predictions of the NLS model. This model regards the thin film as a collection of independent regions with distributed switching times, and their distribution originates from the fluctuation of the local electric field, which is caused by grain boundaries, defects, and multiphase coexistence. Cristóbal Alessandri et al. successfully extracted the minimum switching time *τ*_*∞*_ (~ 236 ns), the activation field *E*_*a*_ (~ 2.4 MV cm^−1^), and the statistical distribution f(η) of the local electric field enhancement factor from the experimental characterization of HZO thin films and fitting with the NLS model. This distribution usually conforms to the generalized beta distribution, which directly reflects the inhomogeneity of the thin film [63].

It is worth noting that there are significant differences in the switching dynamics between epitaxial thin films and polycrystalline thin films. Although epitaxial thin films exhibit a higher nucleation density, their switching speed is slower than that of polycrystalline thin films. This highlights the rate-limiting role of nucleation in Hf-FEs switching. However, under high electric fields close to the activation field, the switching dynamics of both thin films show a convergence trend between the NLS and KAI models. This indicates that under high-field driving, the local potential barrier is "smoothed" and the switching approaches a uniform, intrinsic limit [114].

Furthermore, the temporal characteristics of hafnium-based ferroelectric devices, such as wake-up and fatigue, are also closely related to oxygen vacancy migration and its pinning/depinning effect on DWs. Wake-up corresponds to the gradual increase of the switchable region during electric field cycling, which is manifested as the evolution of two independent NLS contributions in dynamics. Fatigue, on the other hand, originates from the pinning of DWs due to defect accumulation, resulting in a decrease in switching speed and a reduction in saturated polarization [115]. The dynamics of these cyclic evolutions can also be described and fitted using phenomenological models based on statistical distributions.

In summary, the integration of macroscopic phenomenological models with first-principles computing and atomistic simulations constitutes a multi-scale framework for understanding the unique switching behavior of hafnium-based ferroelectrics. This framework not only reveals the correlation between its microscopic origins and macroscopic manifestations but also provides critical theoretical tools and practical guidance for optimizing material microstructures, predicting device endurance, and designing reliable operation schemes.

#### Mainstream Theories and Controversies

The physical origin of ferroelectricity in HfO_2_ remains a frontier topic of academic discussion. Currently, several competing theoretical perspectives exist, which collectively constitute a diverse framework for understanding this phenomenon.

Phonon-band-driven mechanism: Starting from the microscopic nature of lattice dynamics, this mechanism posits that soft modes exist in the phonon spectrum of certain crystal phases of HfO_2_, such as the cubic or tetragonal phase. Specifically, the frequency of certain vibrational modes decreases with temperature or stress and tends toward zero, leading to lattice instability and acentric distortion, thereby inducing a ferroelectric phase transition. First-principles computing indicates that doping or applying strain can modulate these soft mode behaviors, enabling the dynamic stabilization of the energetically metastable acentric orthorhombic phase (Pca2_1_) [116]. This mechanism emphasizes the intrinsic origin of ferroelectricity, providing a microscopic theoretical basis for understanding the macroscopic effects of doping and strain. The ferroelectricity observed in the pioneering experiments by Böscke et al. in 2011 can be regarded as a macroscopic manifestation of this mechanism realized in specific materials [35, 117].

Defect-mediated mechanism: Unlike the previous mechanism that focuses on perfect lattices, this mechanism emphasizes the critical role of point defects, especially oxygen vacancies. Oxygen vacancies affect ferroelectricity in two ways: first, as a source of electrostatic fields, their ordering can generate a built-in electric field that induces local polarization; second, as charge traps, their charging and discharging processes can assist or modulate the switching of macroscopic polarization [44, 118]. Experimentally observed "wake-up effect" frequency dependence, and fatigue characteristics strongly point to the dynamic behavior of oxygen vacancies [37]. Computing studies further reveal that the presence of oxygen vacancies can significantly change phase stability and reduce the formation energy of the ferroelectric phase. This mechanism primarily elucidates the dynamic evolution of ferroelectricity, reliability concerns, and its extreme sensitivity to preparation conditions.

Interface and size constraint mechanism: This mechanism starts from the macroscopic thermodynamics of the thin film system, pointing out that at the nanometer scale, the contributions of surface energy and interface energy to the total free energy become crucial. Specific top/bottom electrode materials can alter the relative stability of each phase through interfacial stress, chemical bonding, and interfacial charge. This thermodynamically screens and stabilizes metastable ferroelectric o-phases that are difficult to exist in bulk materials [55]. Epitaxial strain experiments have successfully stabilized novel ferroelectric phases [119], providing direct evidence for this. This mechanism mainly explains why ferroelectricity only appears in ultra-thin films and its strong dependence on electrodes and thickness.

Currently, a single mechanism is insufficient to explain all experimental phenomena. The gradually forming consensus in the field is that the ferroelectricity of HfO_2_ originates from a multi-scale and multi-physical field coupling synergistic process. Intrinsic instability driven by the phonon band constitutes the physical basis for the existence of the ferroelectric phase. Interface and size constraints provide a thermodynamic stabilization pathway at the nanometer scale. Oxygen vacancies dominate the polarization reversal behavior, activation process, and reliability at the kinetic level. Recent studies have clearly indicated that its ferroelectricity is the result of the combined action of "metastable phase stabilization", "defect engineering", and "interface modulation" [3, 53].

### Performance

Hf-FEs thin films achieve high-performance ferroelectric devices compatible with CMOS technology by stabilizing the o-phase. These films offer core advantages, including high remanent polarization, ultrafast polarization switching, and excellent endurance, thus providing a physical basis for novel non-volatile memory technologies.

**Fig. 6:**
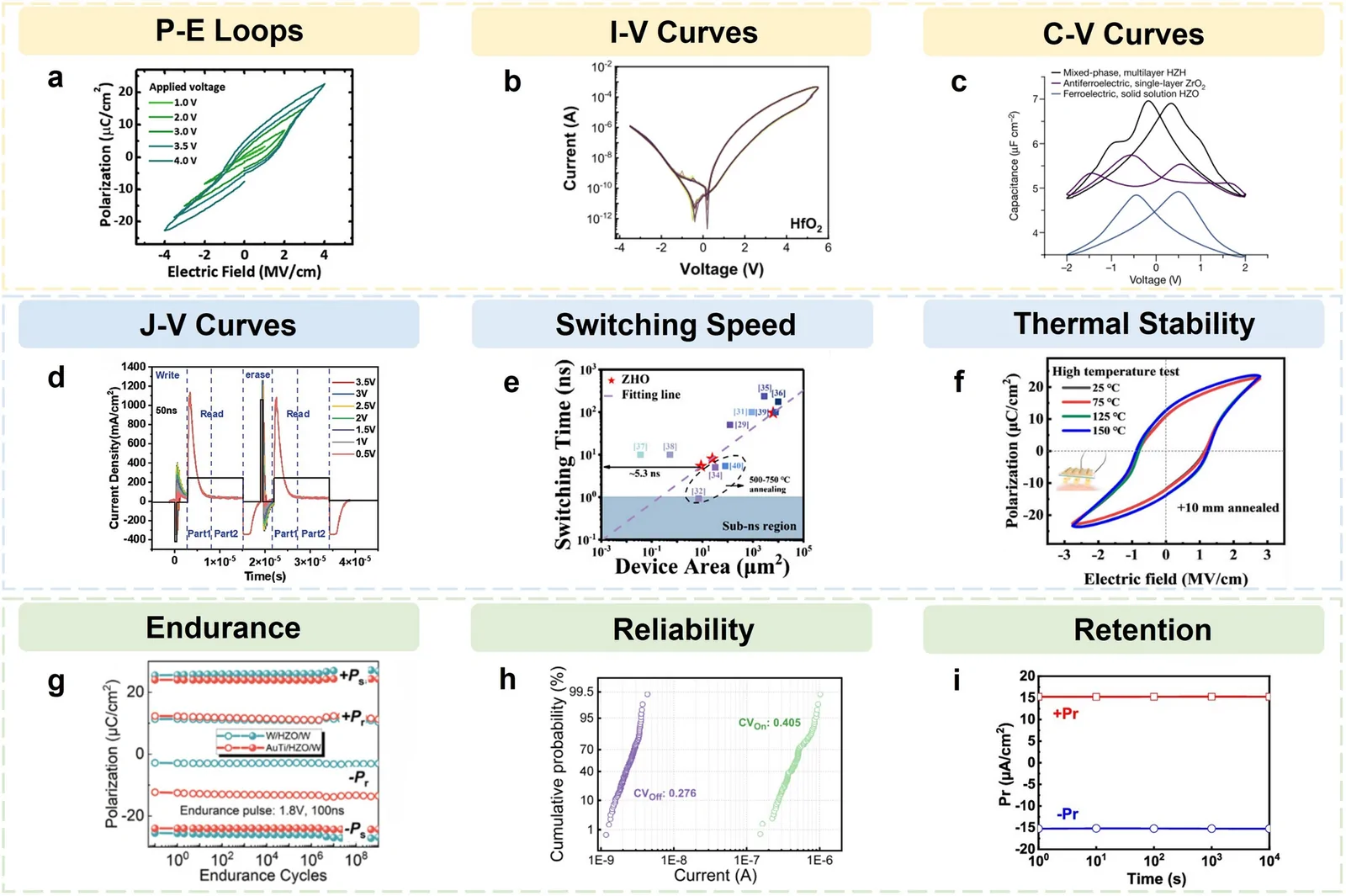
Performance of Hf-FEs devices. **a** P-E hysteresis curve for the Al:HfO_2_ MFM capacitors at different applied voltages [80]. Copyright 2020, Royal Society of Chemistry. **b** I–V characteristics of samples with HfO_2_ obtained by sweeping above the dual-voltage range [120]. Copyright 2024, Elsevier. **c** C-V hysteresis loops for a mixed FE–AFE HZH multilayer [1]. Copyright 2022, Springer Nature. **d** Current density response of the device under different writing voltages [121]. Copyright 2025, Wiley–VCH. **e** Benchmark of switching time of ZHO film versus process temperature [122]. Copyright 2025, American Chemical Society. **f** P–E loops of 10 mm annealed thin film at high temperature [123]. Copyright 2025, Springer Nature. **g** Durance of saturation polarization and remnant polarization for W/HZO/W and Al/HZO/W devices [58]. Copyright 2024, American Chemical Society. **h** On-state and off-state cumulative probability from 10 cycles of 10 cells [124]. Copyright 2024, American Chemical Society. **i** Retention characteristics under ± 3 V [4]. Copyright 2024, American Chemical Society

Regarding basic electrical responses, polarization–electric field loops serve as the core basis for characterizing ferroelectricity, directly confirming the presence of strong ferroelectric polarization in the material. As shown in Fig. 6a, under a driving voltage of ± 4 V, the device exhibits a maximum polarization greater than 20 μC cm^−2^ and a remanent polarization of 10 μC cm^−2^, with the coercive field being less than 1.5 MV cm^−1^, thus meeting the requirements for low-power operation [80]. Beyond the static polarization characteristics, the dynamic current–voltage (*I*–*V*) response is crucial for understanding the read–write mechanism. The *I*–*V* curves reveal transient current peaks associated with polarization switching. As shown in Fig. 6b, the polarization increases with an increasing electric field and decreases with a decreasing electric field, but it does not completely return to its initial state. This characteristic demonstrates the inherent memory function of ferroelectric materials. The results demonstrate that the device exhibits self-rectifying characteristics and resistance switching capabilities, which are extremely beneficial for storage devices in cross arrays by effectively suppressing crosstalk [120]. Capacitance–voltage (*C*–*V*) testing is indispensable for further evaluating the dielectric behavior and operating range of the device. The *C*–*V* response exhibits a symmetrical "butterfly" shape, with the peak of the dielectric constant corresponding to the critical point of the ferroelectric phase transition, thus providing a basis for designing the operating window of the device. As shown in Fig. 6c, the total capacitance of this superlattice is greater than that of traditional anti-ferroelectric ZrO_2_ and ferroelectric Zr:HfO_2_ of the same thickness, further indicating the characteristics of the hybrid ferroelectric-anti-ferroelectric ordering. This observation demonstrates the superior performance of the HfO_2_-ZrO_2_ superlattice stack [1]. Leakage current performance is a key indicator for evaluating the reliability of non-volatile memory devices. Pulsed measurements of leakage current density are employed to assess the read/write functionality, which aids in investigating their storage characteristics. The test results under a single read/write cycle (Fig. 6d) reveal that the read current remains stable across all write voltages. This confirms the data non-volatility and highlights the excellent storage properties of the Hf-FEs devices [121].

In terms of dynamic switching performance, the nanosecond level polarization switching speed is a hallmark advantage of Hf-FEs devices. As reported in Fig. 6e, the HZO device achieved *τ₁* ≈ 5.3 ns when its area was reduced to 9 μm^2^. Furthermore, the switching time of HZO thin film is predicted to potentially be shortened to the sub-nanosecond range, exceeding the performance of traditional PZT devices by 1–2 orders of magnitude [122].

Reliability assessment is a key metric for evaluating the performance of Hf-FEs devices. Firstly, thermal stability ensures device reliability under complex environmental conditions, as shown in Fig. 6f, Ce-doped HfO_2_ thin film at + 10 mm and − 10 mm exhibits exceptional thermal stability. Their ferroelectric performance remains stable even at 150 °C, highlighting their excellent high-temperature tolerance [123]. Secondly, endurance determines the device’s operational lifetime, as illustrated in Fig. 6g, revealing that work function-engineered Au/Ti/HZO/W capacitors possess outstanding stability: negligible degradation of ± *P*_*s*_ (< 5%) and ± *P*_*r*_ (< 7%) is observed after 10^9^ cycles at an electric field of 3 MV cm^−1^. This performance surpasses that of symmetric W/HZO/W devices (with degradations of 8% and 12%, respectively) [58]. Furthermore, uniformity in device performance is crucial for large-scale array integration. Fig. 6h displays the cumulative probability of on- and off-state currents for 10 cells over 10 cycles. The on/off current ratio distribution was obtained from 10 DC cycles of a single FTJ device. The coefficients of variation for the on and off states are 0.405 and 0.276, respectively. These small variations are beneficial for implementing synaptic devices, ensuring operational consistency [124]. Finally, retention testing is central to verifying data non-volatility, as presented in Fig. 6i, a retention time > 10^4^ s is maintained under a ± 3 V bias voltage, confirming their long-term stability for non-volatile memory applications [4].

The reliability of Hf-FEs devices is supported by three pillars: thermal stability, endurance, and uniformity. These pillars are deeply interconnected and exert mutual constraints. Thermal stability serves as the physical foundation for device functionality, determining the intrinsic robustness of the ferroelectric orthorhombic phase under thermal stress. Its quality directly restricts data retention capabilities in high-temperature environments. Endurance reflects the device’s ability to survive under dynamic operation. The accumulation and annihilation of oxygen vacancies at the interface during cyclic polarization directly affect the decay rate of polarization strength and the device’s operational lifetime. Uniformity, as a critical bridge from single devices to large-scale arrays, whose coefficient of variation not only determines the circuit’s design margin and energy consumption efficiency but also restricts the overall system’s reliability ceiling through the "weakest unit" effect. Superior thermal stability provides a foundation for high endurance, while sophisticated interface engineering, such as work function layer design, can improve the uniformity of electrical properties in addition to increasing endurance. Therefore, a true breakthrough in reliability depends on the collaborative optimization of material phase stability, interface defect engineering, and process uniformity control. This approach overcomes the limitations of focusing on a single performance metric and achieves a systematic improvement in the overall device reliability.

### Applications in Memory

Hf-FEs materials possess compelling characteristics, notably their robust polarization switching at the nanoscale, outstanding endurance exceeding 10^10^ cycles, compatibility with mainstream CMOS processes, and tunable coercive field, positioning them as strong candidates for next-generation non-volatile memory. Based on these materials, novel memories using Hf-FEs are continuously being explored [125, 126], such as high-density, low-power ternary content addressable memory (TCAM) and advanced memory applications like 3D ferroelectric NAND (FeNAND) flash memory.

**Fig. 7:**
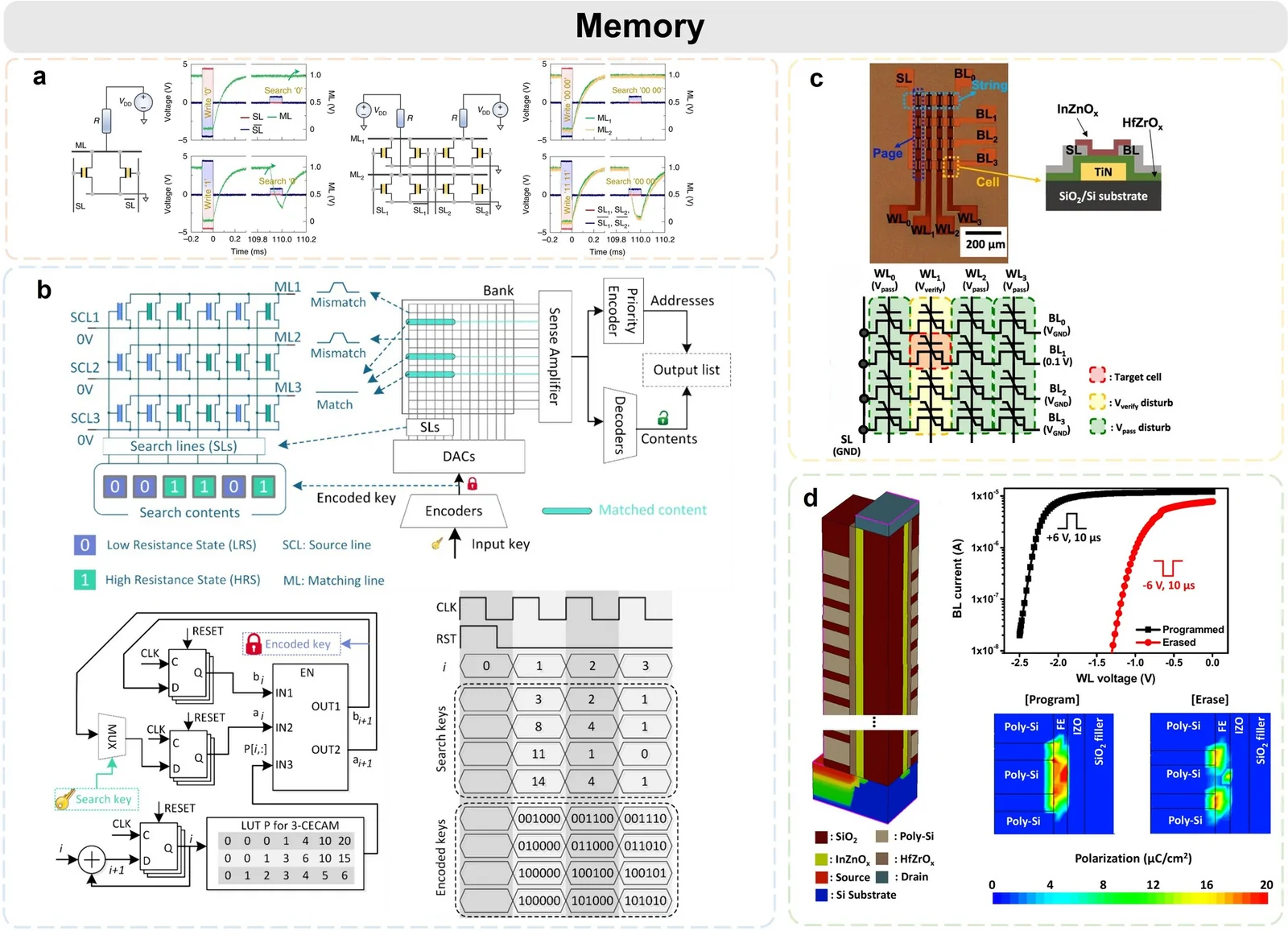
Applications in memory. **a** Operation of a single 2FeFET TCAM cell and 2 × 2 TCAM array [127]. Copyright 2019, Springer Nature. **b** Circuit architecture of CECAM-based search engines [128]. Copyright 2025, American Chemical Society. **c** Optical microscopy image of the 2D FeNAND array and schematic circuit array structure and pulse scheme used for read operation of target cell located at WL1 and BL1 [130]. Copyright 2024, American Chemical Society. **d** Device structure of simulated 3D FeNAND and BL current-WL voltage curves of 3D FeNAND memory cell in erased and programmed states [129]. Copyright 2024, American Chemical Society

TCAM is widely regarded as one of the most promising memory technologies, playing a crucial role in network and computing systems by enabling high-speed search operations [131–133]. Ni et al. proposed utilizing TCAM as an attention memory module, which avoids data movement by directly calculating the distance between query vectors and each stored entry within the memory. They designed a TCAM cell based on two FeFETs and constructed a 2× 2 prototype array (Fig. 7a). Tests in one-shot and few-shot learning tasks demonstrated that this ferroelectric TCAM method reduced the power consumption of a single memory search operation by a factor of 60 and decreased latency by a factor of 2700 [127].

In terms of improving energy efficiency, novel storage architectures such as combinational encoding content addressable memory (CECAM) have emerged in recent years. CECAM outperforms traditional TCAM in terms of content storage density. Figure 7b illustrates a CECAM cell based on ferroelectric HZO FeFET. Unlike traditional TCAM, which requires a pair of complementary switches per bit to store '0', '1', or 'don't care' states, this architecture eliminates the complementary pair structure. Instead, it employs 2N independent switches that enable the combinatorial encoding of any combination of *N* 0s and *N* 1s, thereby overcoming the limitations of bit-by-bit processing. This design, while enabling efficient parallel search, also boasts negligible standby power consumption and significantly reduced dynamic power consumption—particularly in mismatch scenarios [128].

Despite enhanced TCAM technologies, such as CECAM, showcasing the potential of ferroelectric materials in efficient, high-speed associative computing, the escalating demand for storage density driven by big data and AI applications underscores the need for more scalable solutions. Consequently, NAND flash memory architectures, which offer both ultra-high density and cost-effectiveness, have emerged as a significant candidate solution [134–136]. As illustrated in Fig. 7c, 2D FeNAND arrays represent a crucial step in transforming the FeFET concept into practical NAND memory technology. Its structure is similar to that of charge trapping NAND, where each basic unit comprises multiple series-connected FeFETs. These transistors share common source/drain diffusion regions and are individually controlled by separate word lines [130].

However, despite 2D FeNAND demonstrating promising potential in experimental verification, its planar geometry inherently limits its density when scaling to terabit and beyond storage capacities. To overcome this limitation and fully realize the high-density potential of FeNAND, vertical stacking must be achieved through three-dimensional integration. The excellent conformality of atomic layer deposition (ALD) for key materials, such as doped HfO_2_ and TiN, coupled with their robust thermal budget tolerance during the stacking process, makes Hf-FEs exceptionally well-suited for transitioning to a three-dimensional FeNAND architecture.

3D FeNAND employs vertically stacked memory holes or trench arrays. In these high aspect ratio structures, gate-all-around or double-gate configurations are commonly adopted, featuring cylindrical or slit-shaped polysilicon channels surrounded by ferroelectric gate stacks. This multilayer design significantly improves the bit density per unit area. Kim et al. demonstrated Hf-FEs transistors using polysilicon gate electrodes, achieving a large memory window of 2.2 V, a fast switching speed of 10 ns, and a high endurance of 10^7^ cycles (Fig. 7d) [129].

## Neuromorphic Computing Based on Hf-FEs

### Synaptic Plasticity

With the exponential growth of data generation and processing volume, modern computing systems based on the traditional von Neumann architecture have become inadequate. Neuromorphic computing, inspired by the organization and function of the human brain, is widely regarded as a promising next-generation AI technology due to its remarkable biomimetic characteristics in computational parallelism, energy consumption, and system scalability [137]. Given the bio-like device behavior of ferroelectric devices, they are employed to emulate key biological structures in neuromorphic computing, such as synapses and neurons [14, 138–142]. Hf-FEs synaptic devices perfectly meet the core requirements of neuromorphic computing for a hardware platform, owing to their seamless CMOS process integration, emulation of polymorphic behavior, low-power consumption, and non-volatility.

**Fig. 8:**
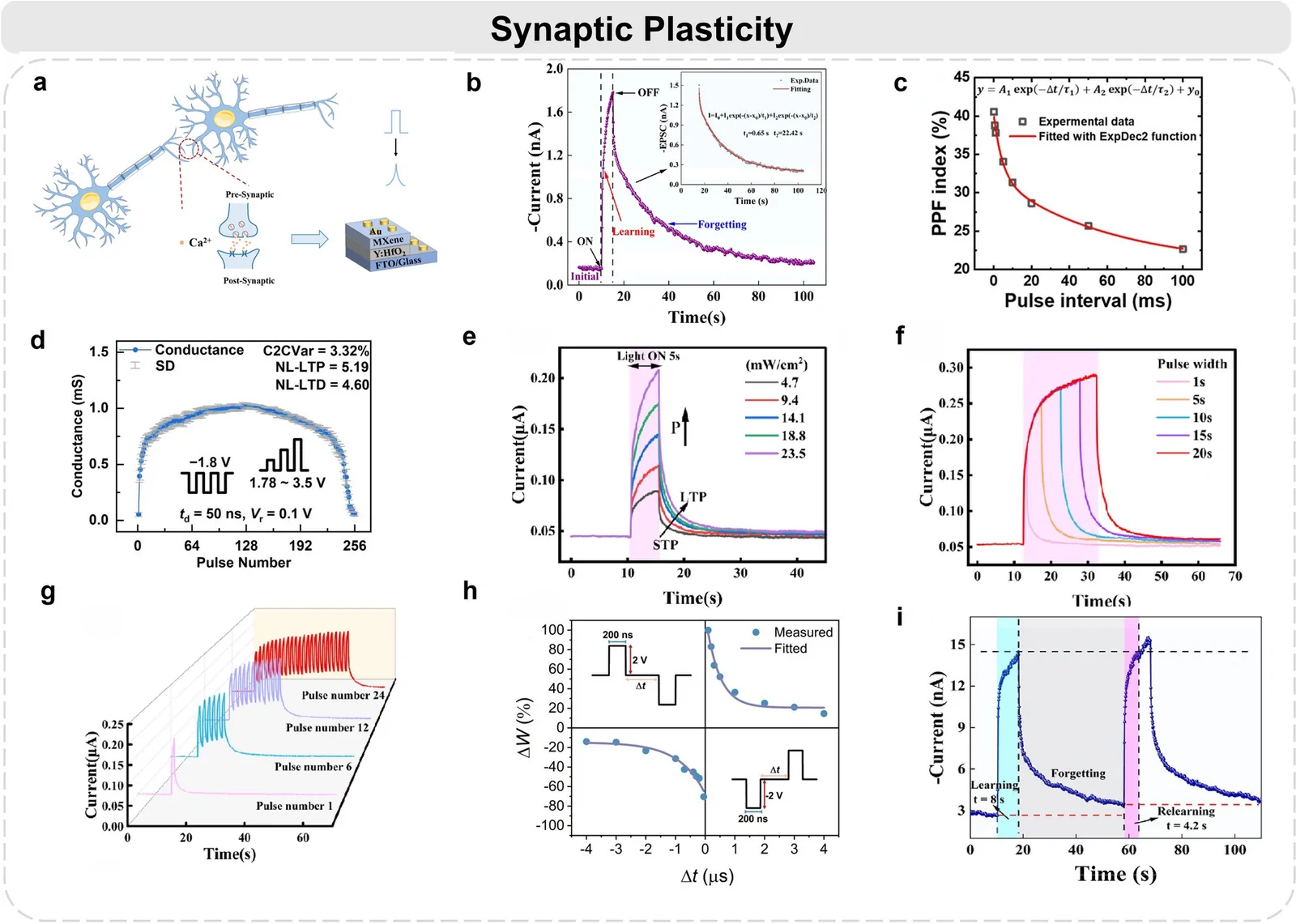
Synaptic Plasticity. **a** Analogy between biological synapses and ferroelectric memristor. **b** EPSC under a single light exposure. **c** PPF index fitted using the double exponential decay function. **d** Corresponding nonlinearity of LTP/LTD and cycle-to-cycle variation obtained using MVPS. **e** SADP. **f** SWDP. **g** SRDP. **h** STDP. **i** Learning-forgetting-relearning process.** a** Reproduced with permission [143]. Copyright 2024, American Chemical Society. **b**, **i** Reproduced with permission [144]. Copyright 2025, Elsevier. **c** Reproduced with permission [145]. Copyright 2024, American Chemical Society. **d** Reproduced with permission [5]. Copyright 2025, American Chemical Society. **e–g** Reproduced with permission [146]. Copyright 2025, Elsevier. **h** Reproduced with permission [147]. Copyright 2024, American Chemical Society

Synaptic plasticity describes the ability to emulate biological synaptic functions. Ferroelectric synaptic devices exhibit inherent structural homology with biological synapses (Fig. 8a). Combined with analogous information processing mechanisms, they enable precise emulation of synaptic plasticity—the neurobiological foundation of learning and memory. The core of implementing biological synapse emulation based on Hf-FEs synaptic devices lies in directly utilizing the non-volatile multilevel polarization states of ferroelectric materials to simulate the continuous change of weights. Through precise doping and interface modulation, Hf-FEs can maintain stable ferroelectricity at an ultra-thin scale, and its polarization vector can undergo partial and continuous reversal under the action of an applied electric field. This characteristic is manifested at the device level as the progressive adjustment of the remnant polarization of the ferroelectric layer, which in turn linearly modulates the channel conductance or tunnel junction resistance. Applying a short voltage pulse that simulates an action potential can induce a small change in the polarization states, thereby achieving gradual changes in conductance similar to long-term potentiation/depression (LTP/LTD). Meanwhile, the high coercive field and optimized endurance ensure the non-volatile retention of weights and reliable cycle tolerance, which is the physical basis for constructing learnable, low-power artificial neural network hardware. Hf-FEs synaptic devices exhibit extremely high endurance, meaning that they can withstand highly frequent weight updates without performance degradation. Furthermore, the ultra-thin nature of Hf-FEs materials allows for lower operating voltages in these synaptic devices, thus reducing computing power consumption. Compared with traditional ferroelectric materials, Hf-FEs materials offer distinct advantages in artificial synapse applications, and Hf-FEs synaptic devices are continually being developed and implemented. Tong et al. reported a ferroelectric memristor with optoelectronic synaptic properties that generate excitatory postsynaptic currents (EPSC) upon stimulation by single UV light pulses (Fig. 8b) [144]. The paired-pulse facilitation/depression (PPF/PPD) phenomenon, a core mechanism of short-term plasticity in biological synapses, is achieved in ferroelectric neuromorphic devices through polarization relaxation dynamics (Fig. 8c) [145]. Repeated pulse trains can induce LTP/LTD in synaptic devices, resulting in persistent conductance increase/decrease that impacts neural network recognition accuracy (Fig. 8d) [5]. Mirroring biological synapses, where memories are classified as short-term (STM) or long-term (LTM) with interconversion potential, pulse amplitude, width, and frequency significantly influence postsynaptic current (PSC) values. These correspond to: spike-amplitude-dependent plasticity (SADP), spike-width-dependent plasticity (SWDP), and spike-rate-dependent plasticity (SRDP). As shown in Fig. 8e-g, pulses modulate device conductance to drive memory transitions between STM and LTM [146]. Spike-timing-dependent plasticity (STDP), a fundamental biological synaptic property, modifies synaptic strength based on the relative timing of pre- and postsynaptic action potentials. This bidirectional plasticity within temporal windows allows synapses to dynamically adjust connection strength according to neuronal activity correlations, a critical neurobiological basis for learning and memory (Fig. 8h) [147]. Furthermore, as biological brains adapt and learn from external stimuli, researchers actively replicate complex synaptic behaviors. Synaptic device experiments now focus on emulating advanced learning capabilities, with Fig. 8i demonstrating learning-forgetting-relearning behavior [144].

Achieving the aforementioned synaptic plasticity behaviors places specific demands on the key performance indicators of Hf-FEs devices. For instance, LTP/LTD requires devices to possess continuous, linear, and symmetric conductance modulation characteristics. This typically necessitates that Hf-FEs thin films exhibit high remnant polarization (*P*_*r*_ > 20 μC cm^−2^) and moderate coercive field (*E*_*c*_ ≈ 1–2 MV cm^−1^) to achieve low-energy consumption (10^6^ cycles) weight updates. For STDP, the switching speed of the device needs to reach the nanosecond level (*τ* < 10 ns) to precisely match the time window of biological pulses. Recent studies have demonstrated that two-terminal AFE HZO capacitors based on work function engineering can achieve stable non-volatility resistive states (retention > 10 years) under programming pulses of ± 2.1 V. They also exhibit excellent cycling endurance (> 10^9^ cycles) and low-energy consumption for synaptic function, down to 245 fJ spike^−1^ [58]. These parameters lay the foundation for realizing low-power, highly reliable analog biological synapse functions. However, compared to ideal synaptic devices, Hf-FEs devices still have room for improvement in terms of linearity of conductance modulation, device-to-device uniformity, and low-voltage operation. These factors directly affect the accuracy and energy efficiency of network training.

### Neuromorphic Computing

The essence of neuromorphic computing lies in emulating the structure and mechanisms of biological neural systems to enable highly energy-efficient and highly parallel information processing. Experimentally validated synaptic plasticity mechanisms, particularly the dynamic behavior of STDP and its capability for multimodal weight regulation, provide a physical basis for constructing large-scale neural networks. These networks, composed of layers of neurons interconnected via synapses, have attracted considerable attention due to their ability to significantly reduce computation time in various classification tasks [148, 149]. In contrast to software-based neural networks operating on conventional silicon chips, which incur high data transfer energy consumption caused by the separation of computing and memory, neuromorphic systems built on Hf-FEs synaptic devices realize in-memory computing. Each ferroelectric synaptic unit performs both storage and computing functions simultaneously, featuring a high degree of parallelism that significantly enhances energy efficiency and computational speed [150–152].

**Fig. 9:**
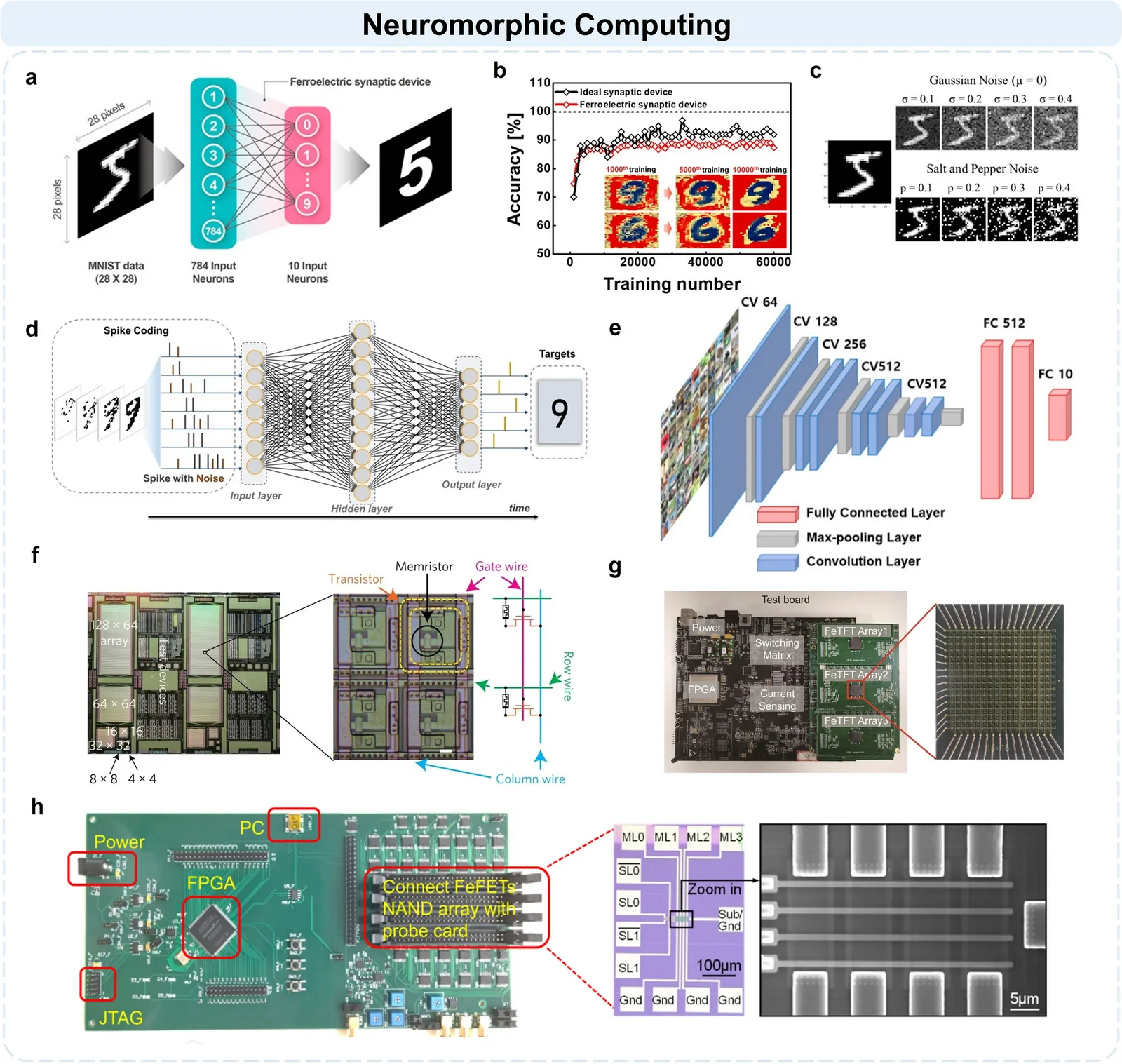
Neural Network. **a** Schematic of the NN and simulated MNIST handwritten data set in the SLP neural network.** b** Pattern recognition accuracy based on both the FeTFT and the ideal synaptic device [61]. Copyright 2021, American Chemical Society. **c** Examples of images with different types and levels of noise. **d** Schematic of the SNN synapse [153]. Copyright 2025, American Chemical Society. **e** Schematic of VGG-11 network [154]. Copyright 2025, Springer Nature. **f** Chip integration based on 1 T-1R memristor crossbars and other test devices [48]. Copyright 2018, Springer Nature. **g** Highly-reliable ferroelectric thin film transistors array for hardware implementation [155]. Copyright 2025, Elsevier. **h** Hardware integration based on 4 × 4 FeFET NAND array and MUSAN method [156]. Copyright 2025, Springer Nature

Neural networks built upon ideal synaptic units facilitate the development of neuromorphic systems that offer high parallel processing capability and excellent energy efficiency. For example, a single-layer perceptron (SLP) was used for MNIST handwritten digit recognition (Fig. 9a). Its input layer consisted of 784 neurons (corresponding to 28 × 28 pixel images), and the output layer contained 10 neurons to represent different digit categories. This configuration achieved a classification accuracy of 90% (Fig. 9b), comparable to theoretical performance [61]. The SLP’s strengths lie in its simple structure and efficient training, rendering it especially well-suited for fundamental pattern recognition tasks. Considering that images in real-world scenarios often contain noise, which can adversely affect classification performance, the noise robustness of the network is critically important. As shown in Fig. 9c, different types and intensities of noise progressively reduce recognition accuracy. Against this background, the spiking neural network (SNN) demonstrates superior adaptability. The SNN model proposed by Heng et al. (Fig. 9d) achieved nearly 98% accuracy in just 50 training epochs and maintained over 90% performance even under strong noise interference. This highlights its practical utility in environments with fluctuating image quality [153]. SNN, through its spike-timing encoding and processing mechanisms, exhibits significant robustness in noisy environments. Furthermore, for more complex image recognition tasks, the VGG-11 network was used to evaluate the applicability of FTJ synaptic units in a convolutional neural network (CNN). The VGG-11 network as shown in Fig. 9e comprises eight convolutional layers, five max-pooling layers, and two fully connected layers. This configuration allows for the effective extraction of multilevel features, making it well-suited for high-precision classification scenarios [154].

In neuromorphic systems based on hafnium ferroelectric devices, different network architectures place varying performance demands on the underlying hardware due to their characteristic differences. Hf-FEs devices must meet these demands through their specific physical properties to achieve efficient computing. The simple structure of the SLP primarily requires non-volatile storage of weights and stable one-write, multiple-read operations. The inherent ferroelectric non-volatility and programmable multilevel polarization states of Hf-FEs devices enable them to reliably store and maintain the weights of the SLP. This characteristic makes the SLP suitable for low-power linear classification and rapid prototyping, despite its limited processing capabilities. The core requirements of a CNN are high-parallelism multiply-accumulate (MAC) operations and efficient energy utilization to process structured data such as images. Hf-FEs devices achieve in-memory computing through crossbar array architecture, completing vector–matrix multiplication (VMM) directly in the analog domain by using Ohm's law and Kirchhoff's laws. This avoids the data transfer bottleneck in the traditional von Neumann architecture and achieves high-energy efficiency inference. The event-driven and asynchronous computing mechanisms of SNNs require hardware synapses to have the ability to respond to precise pulse timing and maintain robustness in noisy environments. The dynamic polarization relaxation characteristics of Hf-FEs devices naturally align with the pulse timing dynamics of SNNs. Experiments have demonstrated that Hf-FEs devices can simulate complex dynamic behaviors such as STDP, which gives them an advantage in time series signal processing and low-power edge computing. The ability of SNNs to maintain high recognition rates in noisy environments is also partly due to the pulse timing-based computing paradigm of Hf-FEs devices. However, realizing these advantages requires devices to have good switching speed consistency and controllable dynamic responses, which places higher demands on materials and interface engineering. Overall, the selection of the network architecture must be deeply matched with the core physical properties of Hf-FEs devices and optimized based on specific application scenarios through trade-offs [11, 157, 158].

At the hardware level, Hf-FEs exhibit substantial potential for implementing non-volatile storage and neuromorphic computing. In contrast to conventional silicon chips based on the von Neumann architecture, which rely on software emulation, incur high-power consumption from data transfer, and face limitations due to the "memory wall", Hf-FEs devices natively support in-memory computing at the physical level owing to their ferroelectric properties. A major strength of these devices is their ability to directly encode neural network weights into the conductance values of devices organized in crossbar arrays, enabling efficient analog VMM to be performed in a single operation. This approach completely bypasses the data movement bottleneck, resulting in orders of magnitude enhancements in both energy efficiency and processing speed. Moreover, such devices can replicate the continuous plasticity observed in biological synapses, facilitate in situ learning, and maintain high compatibility with established CMOS process technologies. These characteristics allow the computing paradigm to be seamlessly incorporated into conventional silicon-based circuits, thereby opening a pathway for heterogeneous integration and co-design of memory, logic, and neuromorphic computing units on a single chip.

Memristor-based computing technology holds great potential in overcoming the so-called "von Neumann bottleneck"inherent in traditional computing architectures. It offers a promising avenue for implementing efficient on-chip learning in various edge intelligence applications [159–162]. Zhang et al. fabricated a full system integrated chip implementing the STELLAR architecture, which integrates multiple memristor arrays with all necessary CMOS peripheral circuits. Using this memristor chip, four continuous learning tasks were implemented. In the MNIST image classification, after three stages of chip learning, the classification accuracies of the training set and the test set on this chip have increased from 8.6% and 8.4% to 94.9% and 92.3%, respectively. At the same time, the energy consumption of the memristor chip is 35 times lower than that of the system based on a digital accelerator [163]. VMM is a core computational task in signal and image processing. VMM operations can inherently be performed in the analog domain using a memristor crossbar array. These memristor crossbar arrays possess advantages such as reconfigurability, reasonable accuracy and precision in physical computing, as well as speed and energy efficiency. Therefore, large-scale memristor crossbar arrays are highly suitable for analog signal and image processing. Figure 9f shows micrographs of two 1T-1R memristor crossbar chips with array sizes ranging from 4 × 4 to 128 × 64 cells. The system has an equivalent 6-bit or 64-level precision and a device yield rate of 99.8%. The energy efficiency is over 119.7 trillion operations per watt, and it uses a 10 ns readout [48]. Ferroelectric thin film transistors (FeTFTs) have attracted extensive attention in in-memory computing applications due to their low-power consumption and potential for monolithic 3D integration. Yang et al. fabricated FeTFT arrays with MFMIS structure units through BEOL processes. They experimentally demonstrated the full hardware implementation of MLP using this array (Fig. 9g), providing a potential hardware solution for efficient in-memory computing systems based on highly reliable FeTFT arrays. The system based on FeTFTs has improved power efficiency by more than two orders of magnitude compared to typical CMOS systems [155]. Figure 9h presents the hardware implementation of the MUSAN edge detector based on a 4 × 4 FeFET NAND array. The system utilizes an FPGA board with an embedded processor to manage data transmission between the FeFET NAND array and the host, as well as to control the array’s peripheral circuits, thereby demonstrating significant energy efficiency advantages [156].

### Image Process

In neuromorphic computing, the core of image processing lies in efficiently executing basic operations such as convolution and feature extraction. The effectiveness of its hardware implementation directly determines the performance of the system when processing tasks like handwritten digit recognition and image classification. Unlike the traditional silicon-based von Neumann architecture, where storage and computing are separated, synaptic devices based on Hf-FEs materials can achieve true "sensing-storage-computing" integration. The physical basis of this advantage is rooted in the unique properties of Hf-FEs. Their CMOS compatibility allows for the construction of high-density, large-scale parallel arrays in back-end processing. Their continuously modulated multilevel polarization states enable the non-volatile storage of neural network weights in the conductance values of the devices in analog form. In specific operations, the input image pixels, acting as voltage signals, are simultaneously applied to the word lines of the arrays. The conductance, representing the weight, of each Hf-FEs device in the arrays generates a corresponding current according to Ohm’s law. These currents are naturally summed on the bit lines according to Kirchhoff’s law, completing the MAC operation between the entire image patch and the convolution kernel in one hardware step. This analog computing method, based on physical laws, fundamentally avoids frequent data transfer between the processor and the memory, greatly reducing data traffic and energy consumption, and achieving extremely high processing speed and low latency due to high parallelism. Therefore, the Hf-FEs image processing system is not only an architectural innovation but also a direct embodiment of its material’s intrinsic properties in the computing paradigm.

**Fig. 10:**
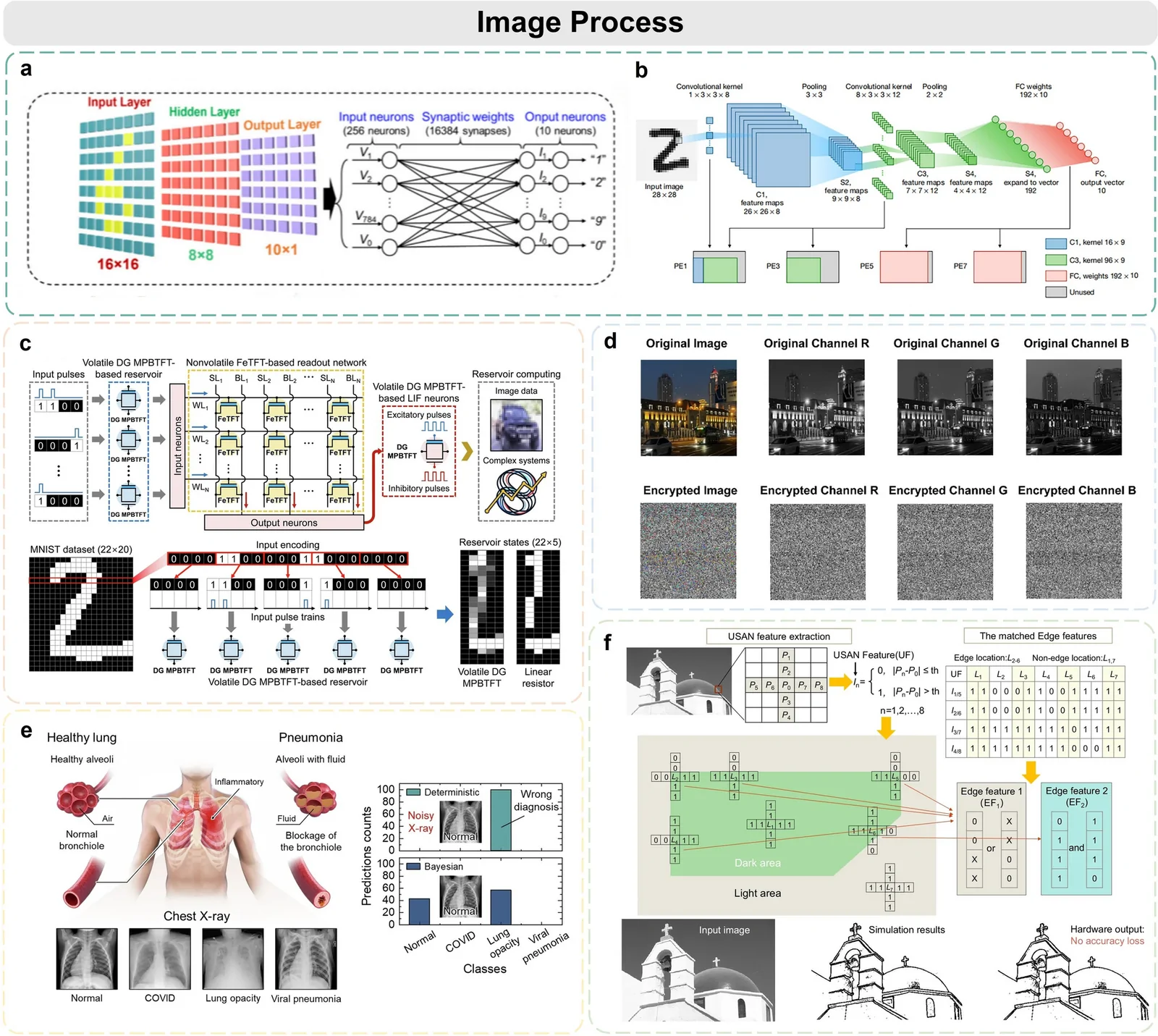
Image process. **a** Illustration of a three-layer ANN with 256 input neurons, 64 hidden neurons, and 10 output neurons for image recognition [58]. Copyright 2024, American Chemical Society. **b** Structure of the five-layer CNN used for MNIST image recognition [164]. Copyright 2020, Springer Nature. **c** MPB-TFT-based ARC system for handwritten digit recognition [165]. Copyright 2024, Springer Nature. **d** Encrypted images corresponding to the original images after encrypting digital images with memristors [166]. Copyright 2025, American Chemical Society. **e** Medical image classification using the 3D FeNAND-based BNN system [167]. Copyright 2025, Springer Nature. **f** Image edge detection based on FeFETs and the MUSAN method [156]. Copyright 2025, Springer Nature

Neural networks are most commonly used for image recognition tasks. Figure 10a, b illustrates two common artificial neural network (ANN) and CNN architectures employed for this task. ANN is a basic fully connected network. For image processing, ANNs require images to be flattened into a one-dimensional vector. This flattening destroys the image’s spatial structure, leads to a large number of parameters, and results in low efficiency, thereby limiting their applicability to simple tasks. CNN is a network specifically designed for images. At its core, a CNN comprises convolutional and pooling layers. These layers automatically extract local image features like edges and textures, which are then combined into higher level representations through a hierarchical structure. Image processing tasks, such as those involving CNNs, demand that hardware possess a high degree of parallelism and low energy consumption for MAC operations. This translates to the following requirements at the Hf-FEs device level: high-density integration (array density > 10^8^ devices cm^−2^), multilevel conductance states (≥ 64 levels) to achieve high-precision weight mapping, low operating voltage (< 2 V) to reduce system power consumption, and good device uniformity.

Recently, Kim et al. reported on a digital recognition analog reservoir computing system based on multi-petal boundary transistors (MPB-TFTs) (Fig. 10c). In this system, FeTFT-based synaptic devices are utilized, with input pulses applied to a physical reservoir constructed from dual-gate MPB-TFTs. The resulting reservoir states are then fed into a readout network, and its output current is transmitted to leaky integrate-and-fire neurons based on dual-gate MPB-TFTs. These neurons generate the final output of the analog reservoir computing system. This system accurately distinguishes 10 types of handwritten digits and achieves a high classification accuracy (~ 90.23%). The MPB-TFT-based physical reservoir consumes 22.5 pJ per input, while the FeTFT-based synaptic device consumes 0.2 pJ per input [165]. In addition to FeTFT-based synaptic devices, ferroelectric memristors also offer significant advantages in neuromorphic computing. Cheng et al. demonstrated the application of HYO-based memristors in image encryption (Fig. 10d). The inherent random distribution of high resistance state and low resistance state within a specific range is leveraged to convert variable resistance values into digital keys for encrypting digital images. As a result, in the encrypted image, the pixel data distribution on the RGB channel becomes uniform, rendering the contained information unrecognizable, which indicates that the image features are effectively hidden [166]. Bayesian neural networks (BNNs) have emerged to address the lack of robust uncertainty quantification in traditional neural networks. BNNs model weights as probability distributions, which enables quantitative uncertainty assessment. This capability makes BNNs particularly suitable for safety–critical applications such as medical diagnosis. Building on this foundation, Song et al. developed a 3D FeNAND-based BNN system for medical data analysis. When classifying chest X-ray images to identify various respiratory diseases, the system achieved a significantly higher diagnostic accuracy through probabilistic predictions compared to other neural networks (Fig. 10e). The classification accuracy of this model can reach approximately 96%, and it has the ability to distinguish different categories even with noisy input data [167]. Edge detection is one of the most critical research focuses in computing vision. Chen et al. reported a low-power edge detection hardware system based on Hf-FEs FeFET (Fig. 10f). In contrast to conventional edge detection methods based on emerging non-volatile memory, a matching unary segment assimilation nucleus method is proposed, bypassing convolution operations. This approach enables efficient image edge detection with low-power consumption (10 fJ per operation) and realizes a hardware system characterized by lossless precision, extremely low-power consumption, and the absence of an analog-to-digital converter, thus providing a viable solution for edge computing [156].

### Logical Operation

Reconfigurable polarization states in Hf-FEs have significantly advanced the development of a new logic-in-memory (LiM) paradigm, which is fundamentally based on realizing basic operations using logic gates. LiM requires devices to have both non-volatile memory and reconfigurable logic functions, with core indicators including nanosecond switching speeds and low operating voltages. Hf-FEs logic devices have achieved switching speeds of 500 ps and endurance of > 10^7^ cycles, supporting dynamic reconfiguration of multi-input logic gates. The energy consumption per logic function can be as low as 1 fJ, which is 1–2 orders of magnitude lower than traditional CMOS logic gates. Traditionally, logic gates, which serve as fundamental units for executing Boolean operations like AND, OR, and NOT, have been primarily realized through combinations of CMOS transistors. While this conventional approach is mature and reliable, it presents challenges such as fixed circuit functionality and constrained energy efficiency, largely due to the data movement bottleneck inherent in the von Neumann architecture. In contrast, Hf-FEs utilize the non-volatility and electrically reconfigurable properties of their ferroelectric domains polarization states to dynamically realize diverse logic functions within a single hardware unit. This capability facilitates in-memory computing operations without the need for data migration, thereby fundamentally circumventing the memory wall limitation and substantially boosting both computing energy efficiency and operational flexibility [168–170].

Precise writing and erasing of logic states hinges on the switching dynamics of ferroelectric polarization. In the context of FTJs, Kho et al. discovered that switching between intermediate resistance states follows a "separated nucleation spot dependent on the polarity of the applied pulse" mechanism. Specifically, positive and negative polarity voltage pulses induce domain switching near the top and bottom electrodes, respectively. This leads to potentially different microscopic domain distributions under the same macroscopic resistance, which is crucial for achieving multi-bit precise programming [172]. As for FeFETs, optimizing the HZO thin film through phase engineering can significantly enhance the accessibility of intermediate polarization states and cumulative polarization characteristics, thereby improving the linearity and number of conductance state modulations [153].

**Fig. 11:**
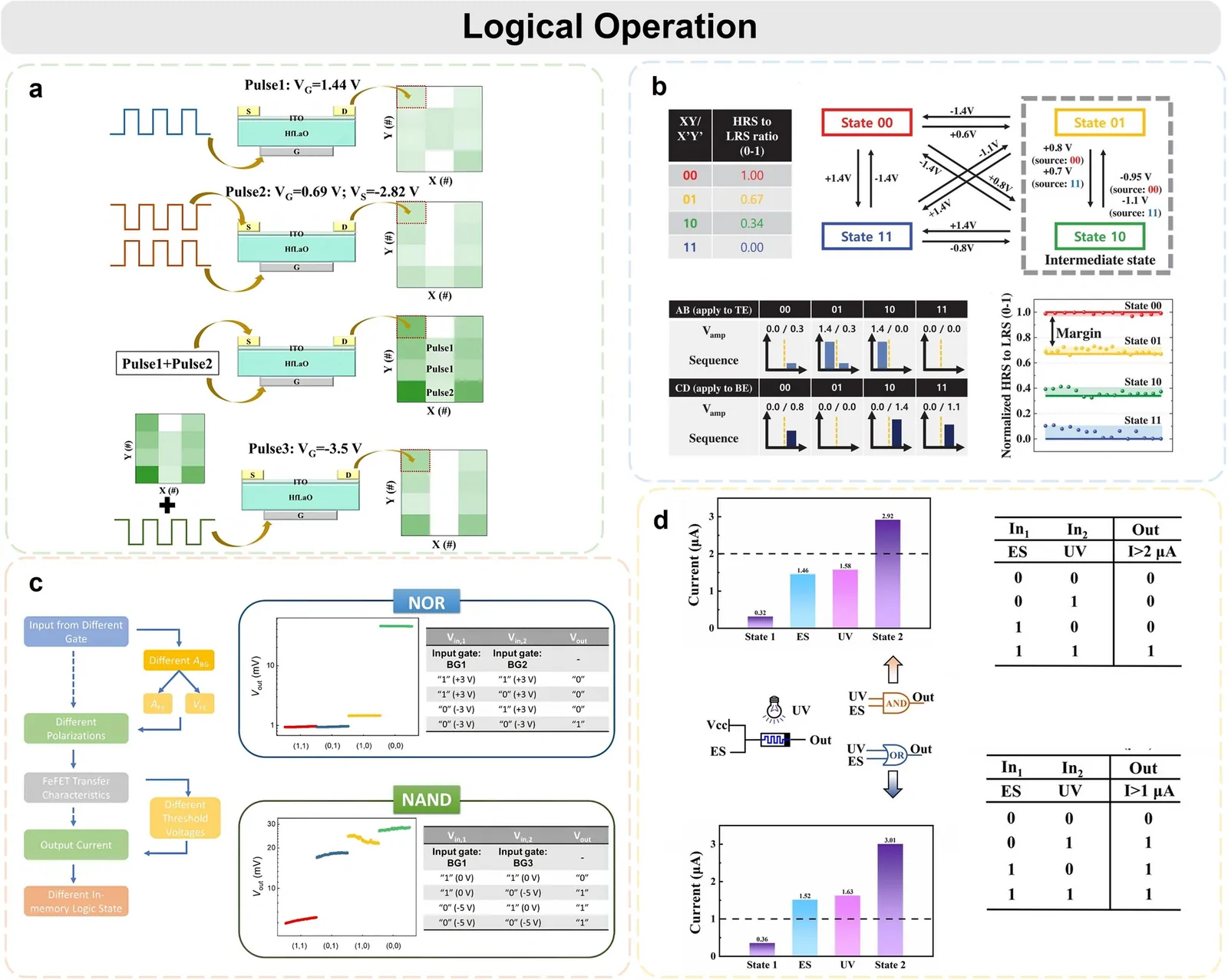
Logical operation. **a** Array-level operation method of "AND " and "OR" ferroelectric logic gates [171]. Copyright 2024, Royal Society of Chemistry. **b** Demonstration of FTJ-based parallel 2-bit LiM [172]. Copyright 2024, Wiley–VCH. **c** Demonstration of LiM using a multi-gate FeFET [153]. Copyright 2025, American Chemical Society. **d** Based on the AND and OR logic operation capabilities of MXene/Y:HfO_2_ devices [143]. Copyright 2024, American Chemical Society

Based on the mechanisms mentioned above, two types of efficient operation paradigms have been developed. In FeFETs/Fe-TFTs, Boolean logic functions can be dynamically reconfigured within a single device through pulse combination strategies. Liu et al. demonstrated an artificial synapse Fe-TFT based on a HfLaO_x_ ferroelectric layer and an ultra-thin ITO channel. By introducing a dual-pulse strategy, they achieved reconfigurable Boolean logic operations within a single Fe-TFT device. The array-level implementation of AND and OR logic gates is shown in Fig. 11a [171]. In FTJs, Kho et al. utilized their V-R timing logic characteristics to achieve parallel 2-bit logic operations on a single device by designing multi-terminal input pulse coding. This approach synchronously generates the output of all 16 Boolean logic functions in just two steps, and the working principle is shown in Fig. 11b [172].

Device level functionalities are driving array-level and system-level applications. Liu et al. have demonstrated image-level parallel AND/OR logic operations based on FeFET arrays, showcasing their potential in analog visual computing. More importantly, through multi-gate FeFET structures, the capacitive coupling effect of different gate regions can be utilized to generate logic outputs corresponding to multiple inputs within a single transistor, thereby enabling reconfigurable logic functions such as inverters, NOR gates, and NAND gates with just a single device, significantly enhancing area efficiency. Xiang et al. reported a multi-gate FeFET suitable for LiM. The operating principle of the multi-terminal FeFET logic gates, along with the corresponding NOR and NAND gate operations, is illustrated in Fig. 11c. This device can be executed within a single clock pulse, and all operation results are stored. The output retention, compared to traditional CMOS-based circuits (the latter requires a constant-voltage power supply to maintain an ideal conductive state), improves the energy efficiency of the device [153]. In addition, Fang et al. have developed an optoelectronic memristor based on MXene materials and Y-doped HfO_2_ thin film. By exploiting the optoelectronic response behavior and different set thresholds, they constructed logic gates driven by optical and electrical inputs. This approach offers a viable pathway for optoelectronic artificial neuromorphic devices. Fig. 11d illustrates the analogy between the optoelectronic memristor used for logic operation and the classical NAND gate and presents experimental demonstrations of AND and OR operations [143].

In conclusion, hafnium-based ferroelectric logic operations technology has progressed beyond fundamental functional verification. Its core lies in leveraging a profound understanding of polarization dynamics and innovative device structures to achieve in-memory computing with high parallelism, reconfigurability, and sensing integration. This offers a critical hardware foundation for developing next-generation energy-efficient intelligent systems.

## Challenges and Prospects

**Fig. 12:**
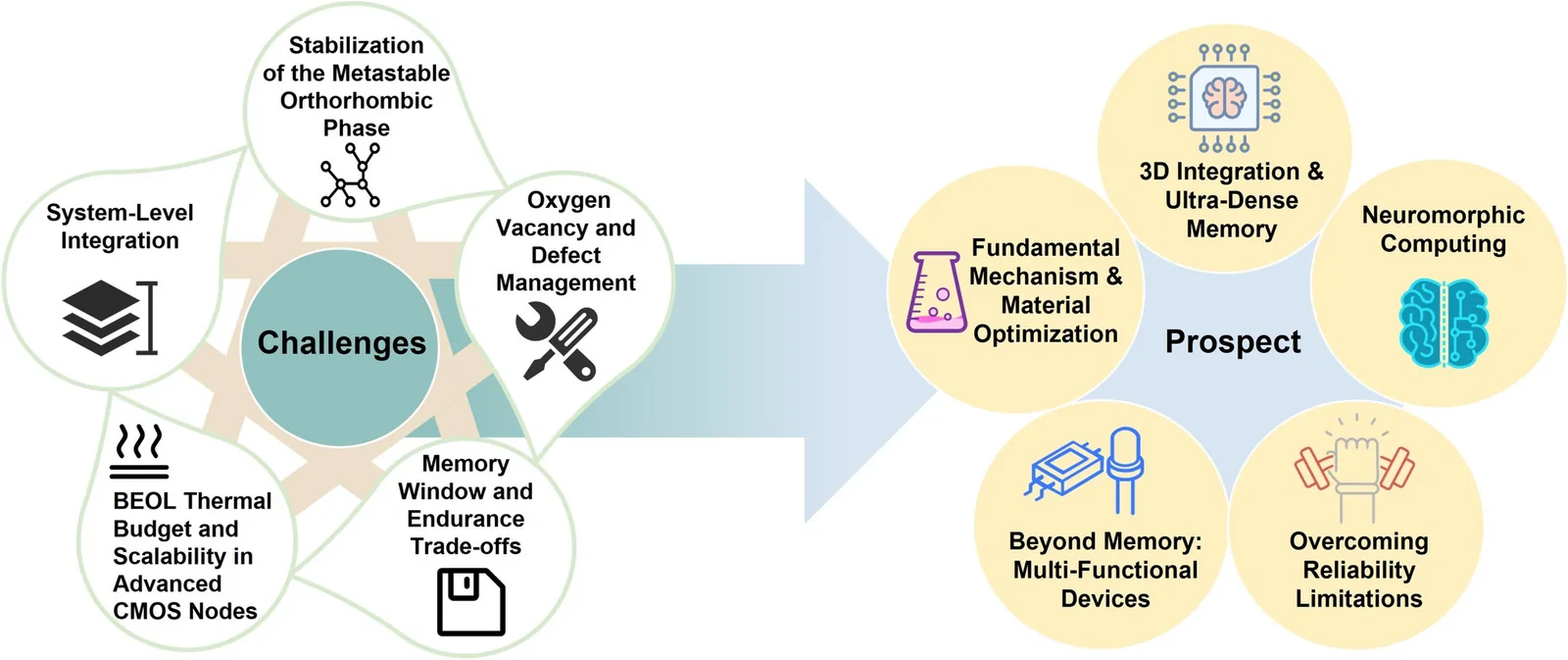
Future prospects and challenges for Hf-FEs

Hf-FEs devices play an important role in modern science and technology, and their rapid development demonstrates great application potential in the fields of next-generation non-volatile memory and neuromorphic computing. Meanwhile, there are still numerous challenges behind these extensive development prospects (Fig. 12).

### Challenges and Recent Strategies

#### Stabilization of the Metastable Orthorhombic Phase

The ferroelectric orthorhombic phase in HfO_2_ is metastable, and its reliable stabilization under CMOS compatible conditions remains challenging. Recent research has primarily focused on external modulation through doping, strain engineering, and interface design. For instance, La-Al co-doping has been shown to synergistically regulate oxygen vacancy and lattice strain, enhancing polarization strength and endurance in 3D FeRAM arrays [71]. Furthermore, region-selective oxygen vacancy engineering via plasma treatment during ALD enables the stabilization of ferroelectric HZO thin films at annealing temperatures as low as 300 °C, significantly improving BEOL compatibility [173]. Another promising approach involves constructing multilayer or superlattice structures; for example, inserting HLO subnanolayers in HZO enhances interfacial polarization coupling and suppresses phase degradation [72]. While these methods improve thermal stability and endurance, they often increase process complexity and may introduce variability in ultra-thin films.

#### Oxygen Vacancy and Defect Management

Oxygen vacancies play a dual role in Hf-FEs: they can stabilize the ferroelectric orthorhombic phase, but an excess of, or mobile, oxygen vacancies can lead to reliability issues such as fatigue, imprint effect, and retention degradation. Recent studies have shown that active control of oxygen vacancies can simultaneously improve device performance and stability. For example, in anti-ferroelectric HZO energy storage capacitors, controlling the oxygen vacancy concentration by tuning the O_2_ flow rate during the sputtering process can optimize the tetragonal/orthorhombic phase ratio, thereby significantly improving the energy storage density (86.3 J cm^−3^) and efficiency while maintaining a cycle life of more than 10^9^ cycles [174]. This demonstrates that controlled oxygen defects can enhance electrical performance while promoting the formation of the target phase without compromising cyclic reliability.

Beyond oxygen vacancy modulation, nitrogen doping is also an effective interface passivation strategy. Nitridation of the ferroelectric layer/electrode interface in FeFETs can reduce interface trap density and suppress oxygen vacancy migration, thereby improving endurance and stabilizing the memory window [175, 176]. However, precise control of oxygen vacancy concentration and distribution at the nanometer scale remains challenging, especially in ultra-thin films and miniaturized devices, where the stochastic nature of defect behavior can affect device uniformity. Future efforts should combine in situ monitoring with real-time process control, as well as computing guidance, to achieve reproducible oxygen vacancy engineering and promote the development of high-density, high-reliability Hf-FEs devices.

#### Memory Window and Endurance Trade-offs

As device dimensions shrink, the memory window tends to narrow, which limits multilevel operation and sensing margin. Recent innovations, including stacked ferroelectric/dielectric heterostructures and negative capacitance effects, have been developed to amplify the effective memory window. For instance, a fully BEOL compatible HZO/IGZO FeFET with an engineered gate stack has achieved an ultra-high memory window of approximately 10 V and an endurance of over 10^9^ cycles through interface dipole engineering and optimized annealing at 300 °C [173]. Dynamic refresh circuits have also been integrated into FeRAM arrays to detect and mitigate fatigue, improving cycle endurance by orders of magnitude [87]. However, simultaneously achieving a high memory window, high endurance, and low operating voltage remains a critical challenge for ultra-dense memory arrays.

#### BEOL Thermal Budget and Scalability in Advanced CMOS Nodes

While Hf-FEs exhibit promising CMOS compatibility, their integration into advanced technology nodes presents intertwined challenges of scalability and BEOL thermal budget incompatibility. The crystallization of ferroelectric HfO_2_ typically requires annealing temperatures above 450 °C, which directly conflicts with the thermal budget limit (< 400 °C) of modern CMOS BEOL processes. To address this, low‑thermal‑budget crystallization strategies have emerged as critical enablers. Recent studies have achieved effective stabilization of the ferroelectric phase in processes below 400 °C through strategies such as plasma-enhanced atomic layer deposition (PE-ALD), ultrafast thermal processing, and material/interface engineering. PE-ALD technology enables direct deposition and crystallization of HZO thin films at 300 °C using highly reactive plasma, resulting in high remanent polarization (*2P*_*r*_ ≈ 20–40 μc cm^−2^) and excellent endurance (> 10^10^ cycles) [177]. Material and interface modulation represents another effective approach. By optimizing the Zr/Hf ratio, ferroelectric ZrHfO_x_ thin films can be obtained after deposition at 280 °C without annealing, exhibiting a high *2P*_*r*_ of up to 30 μC cm^−2^ and an ultra-wide temperature operating range (4.55–473 K) [122]. Employing low thermal expansion coefficient electrodes, like Mo, can also induce interface stress, reducing the crystallization temperature of HZO to 300 °C and achieving a 10 V memory window [178]. Beyond thermal constraints, further scaling of Hf‑FEs devices encounters fundamental material‑level limits. As film thickness approaches 3 nm, the depolarization field intensifies and the stability of the orthorhombic phase becomes critically sensitive to interfacial strain and defect distributions. Moreover, achieving high‑density 3D integration, essential for next‑generation FeNAND and neuromorphic crossbars, demands conformal deposition of ferroelectric layers in high‑aspect‑ratio structures, a task that remains challenging for conventional ALD due to step‑coverage and compositional‑uniformity issues. Variability both within‑device and between devices also escalates with scaling, imposing stringent requirements on process control and circuit‑level compensation techniques.

Moving forward, the co‑design of novel device architectures with BEOL‑compatible low‑thermal‑budget processes will be essential to fully exploit the scalability of Hf‑FEs. Concurrently, efforts must focus on improving process stability, large‑area uniformity, and systematic integration schemes to facilitate the adoption of ferroelectric HfO_2_ in next‑generation memory and computing chips.

#### System-Level Integration

Beyond individual device performance, Hf-FEs' practical application in functional circuits faces critical system-level obstacles. Suppressing crosstalk in high-density crossbar arrays is essential to reduce sneak path currents, which degrade read/write accuracy in memory and synapse arrays. Heterogeneous integration compatibility between novel Hf-FEs devices and traditional CMOS logic requires collaborative optimization of process flows, thermal budgets, and interconnect schemes to ensure hybrid system performance and yield. For reliable neuromorphic computing, robust fault-tolerance mechanisms must be designed to mitigate inherent device-to-device and cycle-to-cycle variability of Hf-FEs synapses. Addressing these challenges demands a holistic, collaborative design approach spanning materials, devices, circuits, and systems.

### Prospects

1. Fundamental mechanisms and material optimization: The atomic-scale origin of ferroelectricity in HfO_2_ remains unclear, particularly regarding the stabilization pathway of the non-centrosymmetric o-phase. Through advanced TEM studies combined with molecular dynamics, nucleation dynamics are expected to be resolved at sub-angstrom resolution, which will guide doping engineering to modulate the coercive field and switching uniformity.
2. 3D Integration and ultra-dense storage: Leveraging ALD-based conformal three-dimensional stacking technology, Hf-FEs have pioneered the next-generation ultra-dense memory, such as 3D FeFET / FeRAM arrays with deep trench capacitors. It is anticipated to achieve more comprehensive performance breakthroughs in areas like storage capacity, read/write speed, and power consumption balance in the future.
3. Neuromorphic computing: In the field of neuromorphic computing, FET-based devices using fluorite-structured HZO materials have garnered significant attention. Anti-ferroelectric FETs, by virtue of their intrinsic properties, have emerged as promising candidates for artificial neurons. This breakthrough establishes a foundation for developing highly integrated artificial biomimetic nervous systems, offering the potential to achieve low-power and high-speed neuromorphic devices.
4. Overcoming reliability limitations: To address the wake-up, fatigue, and imprint issues in Hf-FEs, methods such as interface dipole engineering and work function tuning have been continuously proposed. In the future, these solutions are expected to enable devices to achieve ultra-high endurance cycling and longer data retention, thereby establishing a solid reliability foundation for the widespread application of hafnium-based ferroelectric devices.
5. Beyond memory: Multi-functional devices: The piezoelectric properties of hafnium-based materials enable their utilization in microelectromechanical systems resonators, which can function as 5G/6G radio frequency filters operating within the FR3 band; their pyroelectric effect allows applications in energy harvesting and storage. Within magnetoelectric systems, HfO_2_-based multiferroic materials hold promise for implementation in spin–orbit torque devices.

## Conclusion

Compared to other traditional ferroelectric materials, Hf-FEs materials exhibit good CMOS compatibility and excellent thickness scalability, addressing the demand for continuous device miniaturization. Furthermore, their higher endurance is crucial for memory and neuromorphic computing applications that require frequent writing and erasing, thereby significantly improving device lifetime and reliability. Due to these unique intrinsic advantages, Hf-FEs materials are driving innovation and development in various fields, from ultra-dense non-volatile memory to energy-efficient neuromorphic computing hardware. This paper comprehensively reviews the material system, device structure, and underlying mechanisms of Hf-FEs materials and summarizes their applications in memory, synaptic plasticity, image processing, and logic operations. The importance of Hf-FEs is highlighted through their application in cutting-edge fields, specifically in neural networks and hardware integration designed using these devices. Finally, the challenges and prospects of Hf-FEs devices for future applications in non-volatile memory and neuromorphic computing are thoroughly discussed.
